# Nuclear RNA Decay Pathways Aid Rapid Remodeling of Gene Expression in Yeast

**DOI:** 10.1016/j.molcel.2017.01.005

**Published:** 2017-03-02

**Authors:** Stefan Bresson, Alex Tuck, Desislava Staneva, David Tollervey

**Affiliations:** 1 Wellcome Trust Centre for Cell Biology, University of Edinburgh, Michael Swann Building, King’s Buildings, Edinburgh EH9 3BF, Scotland; 2 Friedrich Miescher Institute for Biomedical Research, Maulbeerstrasse 66, 4058 Basel, Switzerland; 3 European Molecular Biology Laboratory, European Bioinformatics Institute (EMBL-EBI), Wellcome Trust Genome Campus, Hinxton, Cambridge CB10 1SD, UK

**Keywords:** RNA polymerase II, RNA exosome, RNA surveillance, gene expression

## Abstract

In budding yeast, the nuclear RNA surveillance system is active on all pre-mRNA transcripts and modulated by nutrient availability. To test the role of nuclear surveillance in reprogramming gene expression, we identified transcriptome-wide binding sites for RNA polymerase II and the exosome cofactors Mtr4 (TRAMP complex) and Nab3 (NNS complex) by UV crosslinking immediately following glucose withdrawal (0, 4, and 8 min). In glucose, mRNA binding by Nab3 and Mtr4 was mainly restricted to promoter-proximal sites, reflecting early transcription termination. Following glucose withdrawal, many growth-related mRNAs showed reduced transcription but increased Nab3 binding, accompanied by downstream recruitment of Mtr4, and oligo(A) tailing. We conclude that transcription termination is followed by TRAMP-mediated RNA decay. Upregulated transcripts evaded increased surveillance factor binding following glucose withdrawal. Some upregulated genes showed use of alternative transcription starts to bypass strong NNS binding sites. We conclude that nuclear surveillance pathways regulate both positive and negative responses to glucose availability.

## Highlights

• Changes in nuclear surveillance factor binding very rapidly follow nutritional shift
• Downregulated genes frequently show strongly increased surveillance factor binding
• Upregulated genes are protected against elevated surveillance factor binding
• The behavior of functionally related genes indicates posttranscriptional coregulation

## Graphical Abstract

**Figure:**
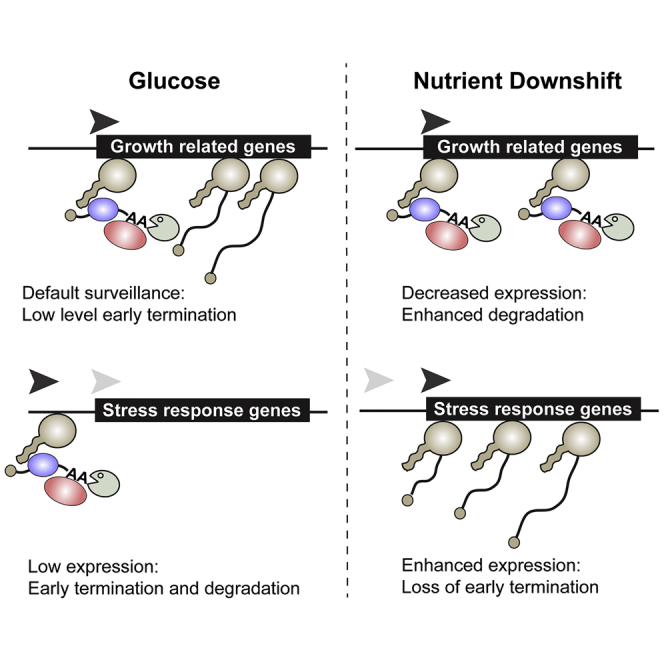

## Teaser

All organisms must rapidly respond to changes in nutrient conditions. We report here that specific changes in nuclear RNA degradation rates help rapidly reprogram gene expression in yeast, acting both positively and negatively. These results identify nuclear mRNA degradation as an actively regulated step in gene expression.

## Introduction

In eukaryotes, a substantial fraction of transcription initiation by RNA polymerase II (Pol II) occurs promiscuously, without regard for functional transcription units (Porrua and Libri, 2015). Left unchecked, the widespread synthesis of nonfunctional RNAs could interfere with normal gene expression. Consequently, cells have evolved sophisticated nuclear RNA degradation pathways to specifically limit the accumulation of such transcripts. In the budding yeast *Saccharomyces cerevisiae*, nuclear RNA decay is largely mediated by the exosome, a conserved protein complex with both 3′ exonuclease and endonuclease activity (Mitchell et al., 1997). The exosome participates in the processing of stable noncoding RNAs such as small nucleolar RNAs (snoRNAs) and degrades cryptic unstable transcripts (CUTs) that can generally be detected only when the nuclear exosome is partially inactivated (Davis and Ares, 2006). The exosome is directed to target transcripts by sets of nuclear and cytoplasmic cofactors. Key nuclear cofactors include the Trf4/5-Air1/2-Mtr4-polyadenylation (TRAMP) complex (LaCava et al., 2005, Vanácová et al., 2005, Wyers et al., 2005) and the Nrd1-Nab3-Sen1 (NNS) complex (Arigo et al., 2006a, Arigo et al., 2006b, Conrad et al., 2000, Mayer et al., 2012, Schulz et al., 2013, Steinmetz et al., 2001). The TRAMP complex consists of three proteins: a poly(A) polymerase (either Trf4 or Trf5), a zinc knuckle protein (Air1 or Air2), and the RNA helicase Mtr4. Trf4 or Trf5 adds a short oligo(A) tail to the 3′ end of the RNA, and this primes the transcript for degradation or processing by the nuclear exosome, assisted by the helicase activity of Mtr4. In the NNS complex, Nrd1 and Nab3 are sequence-specific RNA binding proteins (Conrad et al., 2000), whereas Sen1 (Senataxin in humans) has RNA helicase activity (Porrua and Libri, 2013).

In budding yeast, alternative transcription termination pathways substantially affect the stability of Pol II-derived transcripts (Mayer et al., 2012, Tuck and Tollervey, 2013). In the canonical pathway, protein coding transcripts are cleaved downstream of the open reading frame and polyadenylated, giving rise to stable mRNAs. Noncoding RNAs (ncRNAs) of the stable unannotated transcript (SUT) class are also terminated by this mechanism (Porrua and Libri, 2015, Tuck and Tollervey, 2013). Alternatively, RNAs may be targeted early in the transcription cycle by the NNS pathway (Arigo et al., 2006a, Arigo et al., 2006b, Conrad et al., 2000, Mayer et al., 2012, Schulz et al., 2013, Steinmetz et al., 2001). Many transcripts terminated by NNS, including CUT ncRNAs, are then degraded by TRAMP and the nuclear exosome complex.

NNS recognizes target RNAs through interactions with both the nascent transcript and elongating Pol II (Conrad et al., 2000). Nrd1 and Nab3 bind RNA consensus sequences (UGUA and UCUU, respectively) (Creamer et al., 2011, Wlotzka et al., 2011) and phosphorylated serine 5 (Ser5-P) residues within the repetitive C-terminal domain (CTD) of Pol II (Gudipati et al., 2008, Kubicek et al., 2012, Vasiljeva et al., 2008). Ser5 phosphorylation predominates during early transcription but is rapidly lost after the first few hundred nucleotides in humans and after ∼100 nt in yeast (Milligan et al., 2016). Nrd1 binding to the CTD is inhibited by tyrosine 1 (Tyr1) phosphorylation, which is lowest near the transcription start site but increases thereafter (Mayer et al., 2012, Milligan et al., 2016). The requirement for both phosphorylated Ser5 and dephosphorylated Tyr1 restricts NNS binding to short, noncoding RNAs or the 5′ end of longer protein coding transcripts. When bound to the nascent transcript, Nrd1 and Nab3 recruit the helicase Sen1, which may directly promote dissociation of the Pol II elongation complex from the DNA, although the mechanism is unclear. The NNS complex also directs premature transcription termination and decay of some mRNA genes, including *NRD1* (Arigo et al., 2006a, Steinmetz et al., 2001), *URA2*, *URA8*, *ADE12* (Kuehner and Brow, 2008, Thiebaut et al., 2008), and *FKS2* (Kim and Levin, 2011). Nrd1 and Nab3 bind many other protein coding transcripts (Creamer et al., 2011, Schulz et al., 2013, Webb et al., 2014), but the functional significance of this has been unclear. In addition to its role in termination, NNS also recruits the TRAMP complex to RNAs through a direct interaction between Nrd1 and the Trf4 subunit (Tudek et al., 2014).

Previous analyses indicated that the activity of nuclear surveillance in degrading unspliced pre-mRNAs was modulated by carbon source availability (Bousquet-Antonelli et al., 2000). We hypothesized that the nuclear surveillance pathway participates more generally in reprograming gene expression under conditions of rapidly changing nutrition, which will very frequently be encountered by yeast growing in the environment. When growing in medium containing both glucose and an alternative carbon source, cells preferentially metabolize glucose. Following glucose exhaustion, yeast undergoes major metabolic adaptation, a phenomenon known as diauxic shift. During this time, cells rapidly shut down the expression of growth-related genes to conserve energy while activating the expression of stress response and various metabolic genes. This requires genome-wide changes in both transcription and translation (Ashe et al., 2000, DeRisi et al., 1997, Zid and O’Shea, 2014). Overall transcription is decreased by ∼90% (Jona et al., 2000), and translation is almost entirely halted (Ashe et al., 2000). However, the role of nuclear RNA decay during glucose withdrawal is less clear.

To assess the importance of NNS and TRAMP in the rapid reprogramming of nuclear gene expression during diauxic shift, we used UV crosslinking and analysis of cDNAs through high-throughput sequencing (CRAC). We identified the genome-wide binding sites of Pol II, Nab3, and the TRAMP component Mtr4 as well as RNA abundance after shifting cells from glucose to an alternative carbon source. We find that NNS and TRAMP target hundreds of growth-related genes minutes after glucose withdrawal. In glucose-replete medium, a subset of mRNAs show high levels of Nab3 and Mtr4 binding that is rapidly lost following glucose withdrawal, presumably as a means of increasing gene expression. We also find that genes involved in stress response are transcriptionally induced but escape targeting by Nab3 and Mtr4. Together, our results show that NNS and TRAMP actively participate in remodeling global gene expression in response to changing nutrient conditions.

## Results

**Figure 1:**
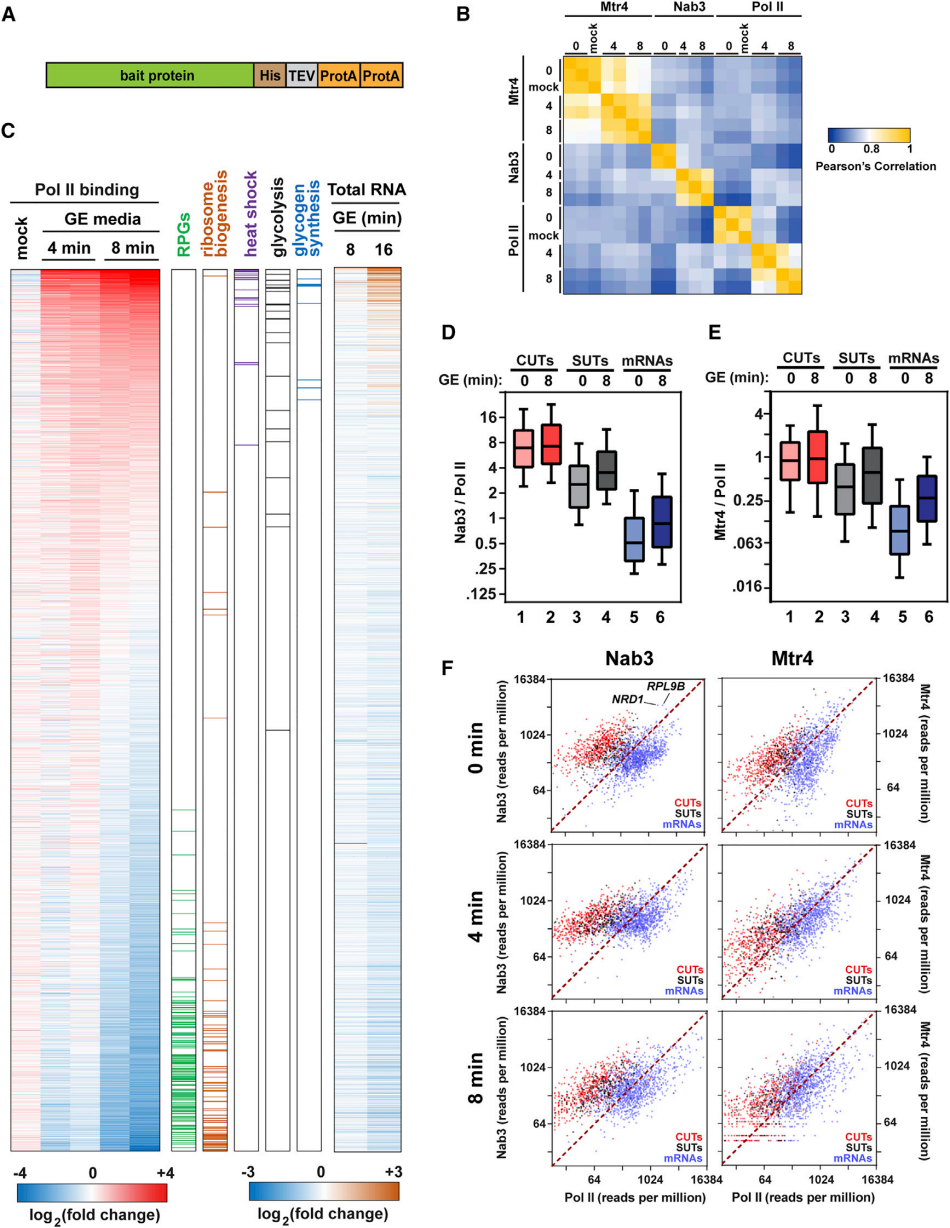
Global Changes in Transcription and Surveillance Factor Binding Following Nutrient Downshift (A) Schematic of HTP-tagged fusion proteins. The HTP tag consisted of His_6_-TEV cleavage site-2X protein A and was fused to the C terminus of the bait protein. (B) Heatmap showing pairwise Pearson correlation coefficients between all CRAC datasets examined in this study, based on the top 2,000 transcribed mRNAs. (C) Heatmap showing changes in Pol II (Rpo21) binding relative to untreated (time 0) cells following mock treatment or growth in GE medium for 4 or 8 min. The top 2,000 transcribed mRNAs, averaged across all time points, are included in the analysis. Red indicates increased levels of Pol II occupancy relative to the glucose control. To the right of the heatmap, genes are colored by GO term. Changes in total RNA levels are shown on the far right. (D and E) CUTs, SUTs, and mRNAs comprise distinct populations when plotted by Nab3:Pol II (D) or Mtr4:Pol II (E) ratios. For each gene, a ratio was calculated by dividing the number of Nab3 or Mtr4 hits per million mapped reads by Pol II hits per million. The boxes show the 25th, 50th, and 75th percentiles, and whiskers show the 10th and 90th percentiles. (F) Nab3 (left) or Mtr4 (right) binding in reads per million (y axis) plotted against Pol II binding in reads per million (x axis) in glucose (top) or 4 min (center) or 8 min (bottom) after shift to GE medium.

To assess the role of nuclear RNA decay in the cellular responses to diauxic shift, we identified transcriptome-wide binding sites for Nab3, Mtr4, and Rpo21 (the catalytic subunit of RNA Pol II) before and after nutrient downshift using the CRAC technique (Granneman et al., 2011). Nab3, Mtr4, and Rpo21 were separately expressed as fusion proteins with a C-terminal HTP tag (His_6_-TEV [Tobacco Etch Virus] protease cleavage site-2X protein A) under the control of their endogenous promoters (Figure 1A). The HTP-tagged proteins were all functional because no growth defects were observed compared with the untagged control strain (Figure S1A, available online). Strains containing the tagged proteins were grown in medium containing 2% glucose until mid-log phase (0.5 optical density 600 [OD_600_]). Cells were quickly filtered and transferred to new medium lacking glucose but containing 2% glycerol and 2% ethanol as alternative carbon sources. Nutrient downshift from glucose to glycerol/ethanol (hereafter called GE) was designed to mimic diauxic shift. After 4 or 8 min in GE medium, cells were UV-irradiated to covalently crosslink RNA:protein (RNP) complexes. As controls, separate cultures were grown in parallel and crosslinked in glucose-replete medium (0 time point) or following filtration and transfer to fresh glucose-containing medium (mock). Cells were lysed, followed by stringent, denaturing, two-step affinity purification of the tagged protein. Bound RNA was partially degraded with RNase and radiolabeled, and 5′ and 3′ linkers were added. Finally, covalent RNA-protein complexes were isolated by SDS-PAGE. Following Proteinase K digestion of the bound proteins, RNA fragments were amplified by RT-PCR. The resulting cDNA was analyzed by high-throughput sequencing and aligned to the yeast genome using Novoalign. Replicate CRAC datasets were acquired for almost all conditions, and most analyses were performed using the average of both replicates. Comparison of replicate CRAC datasets showed good reproducibility (Figure 1B). To complement these datasets, we also performed RNA sequencing (RNA-seq) to analyze steady-state RNA levels 0, 8, and 16 min following nutrient downshift.

We first grouped CRAC reads from different bait proteins by transcript class. As expected, Rpo21 (hereafter called Pol II) was mainly bound to protein-coding RNAs (Figure S1B). Nab3 showed significant binding to CUTs and SUTs, particularly compared with Pol II. Mtr4 bound strongly to rRNA and tRNA, consistent with its role in the processing and nuclear surveillance of these transcripts (de la Cruz et al., 1998, El Hage et al., 2010, LaCava et al., 2005, Vanácová et al., 2005).

### Global Transcription Changes in Response to Glucose Withdrawal

As an initial analysis, we examined Pol II binding for the 2,000 mRNAs with the highest transcription level in glucose-replete medium and 4 or 8 min after shift to GE medium (Figure 1C; Table S1). Individual rows (transcripts) are colored by changes in Pol II binding relative to time 0. Transcripts were sorted by the average fold change across both 8-min replicates, with genes at the top showing increased Pol II binding. Changes in Pol II occupancy were largely consistent with changes in steady-state RNA levels (Figure 1C, right) and previous analyses of RNA abundance following glucose withdrawal (DeRisi et al., 1997, Zid and O’Shea, 2014). This indicated that Pol II binding is a reasonable proxy for transcription rate. Growth-related genes, including ribosomal protein genes (RPGs) and genes involved in ribosome biogenesis, clustered near the bottom, indicating strong transcriptional repression. Conversely, heat shock genes were highly upregulated, consistent with their role in general stress response. Somewhat surprisingly, nearly every gene involved in glycolysis was upregulated (black lines in Figure 1C). This was noted previously for a subset of glycolytic genes (Zid and O’Shea, 2014), but our results indicate that the phenomenon is widespread.

### Global Changes in Binding of Nuclear Decay Factors

Next, we investigated the role of the nuclear quality control machinery in the cellular response to glucose starvation. To this end, we first identified the transcriptome-wide binding sites of the NNS component Nab3 and the TRAMP component Mtr4 prior to glucose withdrawal. Because Nab3 and Mtr4 act on nascent transcripts, we normalized their binding to transcription levels as defined by Pol II occupancy. When compared in this way, CUTs, SUTs, and mRNAs comprised easily distinguishable populations (Figures 1D and 1E, compare columns 1, 3, and 5). These differences were largely lost when using unnormalized data because of the higher average transcription of mRNAs (Figure S1C). CUTs had the highest levels of Nab3 and Mtr4 binding, consistent with the expectation that they are rapidly degraded within the nucleus by NNS and the exosome. By contrast, mRNAs showed relatively low levels of Nab3/Mtr4 binding. SUTs showed intermediate levels of binding to Nab3 and Mtr4 (Figures 1D and 1E), suggesting that they are subject to nuclear quality control in addition to cytoplasmic decay (Porrua and Libri, 2015, Xu et al., 2009).

To view individual genes, we plotted Nab3 and Mtr4 binding against Pol II occupancy for the 2,000 genes showing the highest average levels of Nab3 or Mtr4 binding across both conditions (Figure 1F). Consistent with the genome-wide averages, mRNAs, SUTs, and CUTs largely formed distinct clusters in glucose medium. Nearly all CUTs fell above the y = x line, indicating relatively high levels of Nab3 or Mtr4 binding compared with transcription. Notably, a handful of mRNAs showed CUT-like levels of Nab3 binding (Figure 1F). Among these were the *NRD1* and *RPL9B* transcripts, both of which are known targets of the NNS pathway (Arigo et al., 2006a, Gudipati et al., 2012, Steinmetz et al., 2001). Following nutrient downshift, the sharp differences between mRNAs, SUTs, and CUTs were reduced. Protein-coding genes in particular showed enhanced binding to Nab3 (Figures 1D and 1F) and Mtr4 (Figures 1E and 1F). Conversely, CUTs were similarly bound to Nab3 and Mtr4 before and after glucose starvation. These results indicate that the nuclear RNA surveillance machinery is more active against mRNAs following glucose withdrawal.

**Figure 2:**
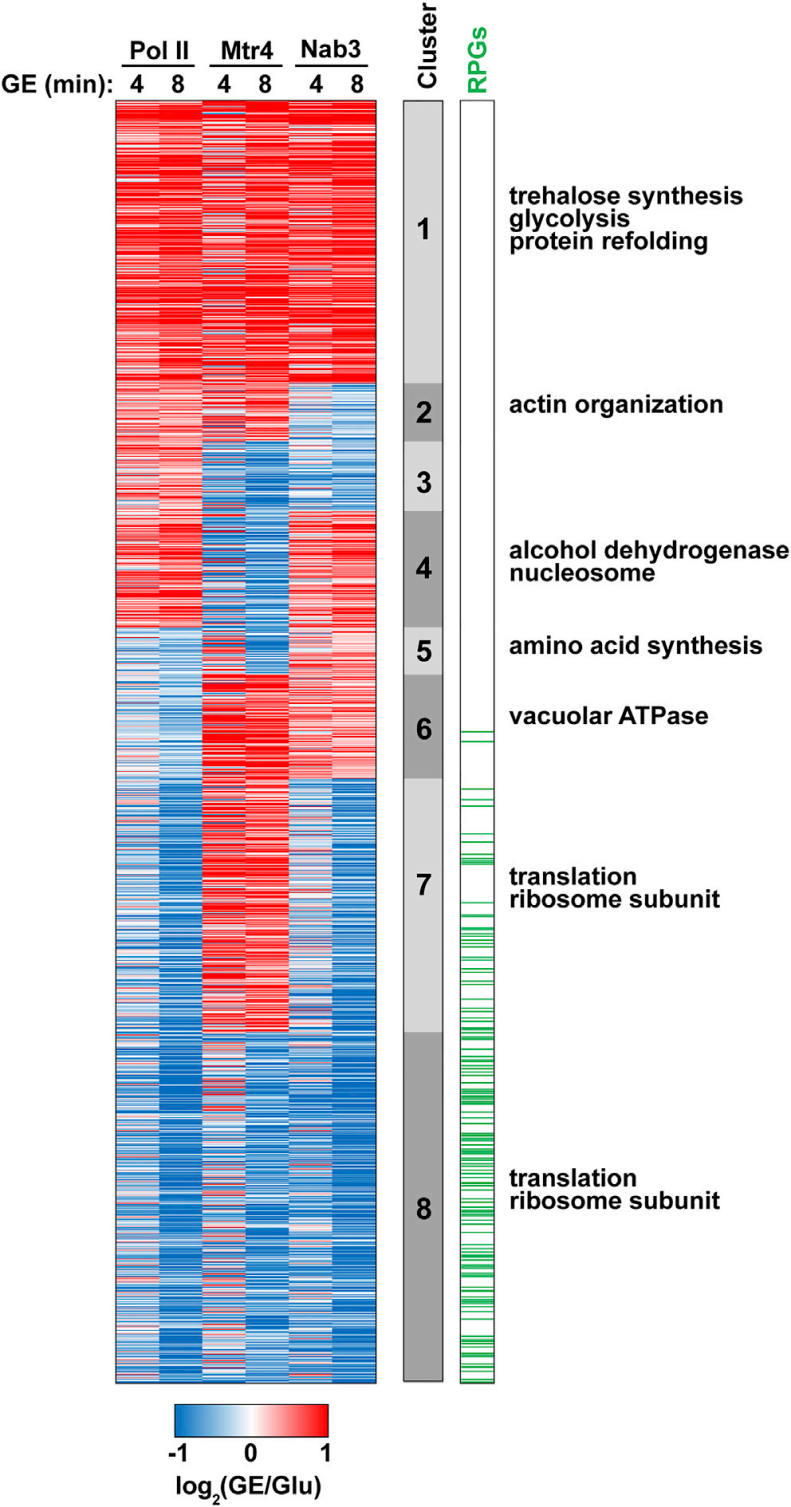
Clustering Analysis of Genes Based on Response to Nutrient Downshift Each row represents a single gene for the 1,570 mRNAs showing high-level Pol II, Mtr4, and Nab3 binding. These were grouped into eight clusters by calculating whether the gene showed increased or decreased binding to Pol II, Mtr4, or Nab3 8 min after glucose starvation. Red indicates high levels of binding relative to glucose conditions. The cluster designations, the distribution of ribosomal protein genes (RPGs) and significant GO terms for each cluster are included at the right of the heatmap.

To further explore this phenomenon, we clustered genes by changes in binding of Pol II and decay factors (Figure 2; Table S2). 1,570 transcripts with good representation in each of the Pol II, Nab3, and Mtr4 datasets were included in the analysis (Figure S2), and eight clusters were generated. For most genes, increased binding to Nab3 or Mtr4 was associated with increased association with Pol II (cluster 1). By default, it might be anticipated that more highly transcribed genes will show greater binding to Nab3 and Mtr4 because more nuclear RNA is available for binding. Likewise, lower transcription levels might result in less Nab3 or Mtr4 binding (cluster 8). However, numerous genes with reduced Pol II occupancy showed increased binding to Nab3 (12% of all genes) or Mtr4 (28%) (clusters 5–7). This subset of genes appeared likely to represent specific targets for Nab3 and/or Mtr4.

Genes from clusters 5 and 6 showed increased targeting by Nab3 despite overall reduced Pol II association. Recruitment of TRAMP to target RNAs can be promoted by direct, physical interactions between Trf4 and Nrd1 (Tudek et al., 2014). Thus, we expected that genes bound by the Nrd1-Nab3 heterodimer should also be bound by the TRAMP component Mtr4. Consistent with this, most cluster 5 and 6 transcripts (86%) also showed increased binding to Mtr4 at either 4 or 8 min (Figure 2). To assess the biological significance of these results, we performed Gene Ontology (GO) term analysis. These Nab3 and Mtr4 targets were enriched for components of the vacuolar ATPase (Figure 2; Table S3), which uses ATP to generate a proton gradient across the vacuolar membrane. We also identified a number of genes positively regulating the cell cycle (e.g., *DBF4*) or involved in amino acid synthesis (e.g., *SAM4*). Overall, the target list (Table S2) suggests that growth-related genes are preferentially targeted by the nuclear decay machinery during glucose starvation.

### Altered Targeting of NNS and TRAMP during Nutrient Downshift

**Figure 3:**
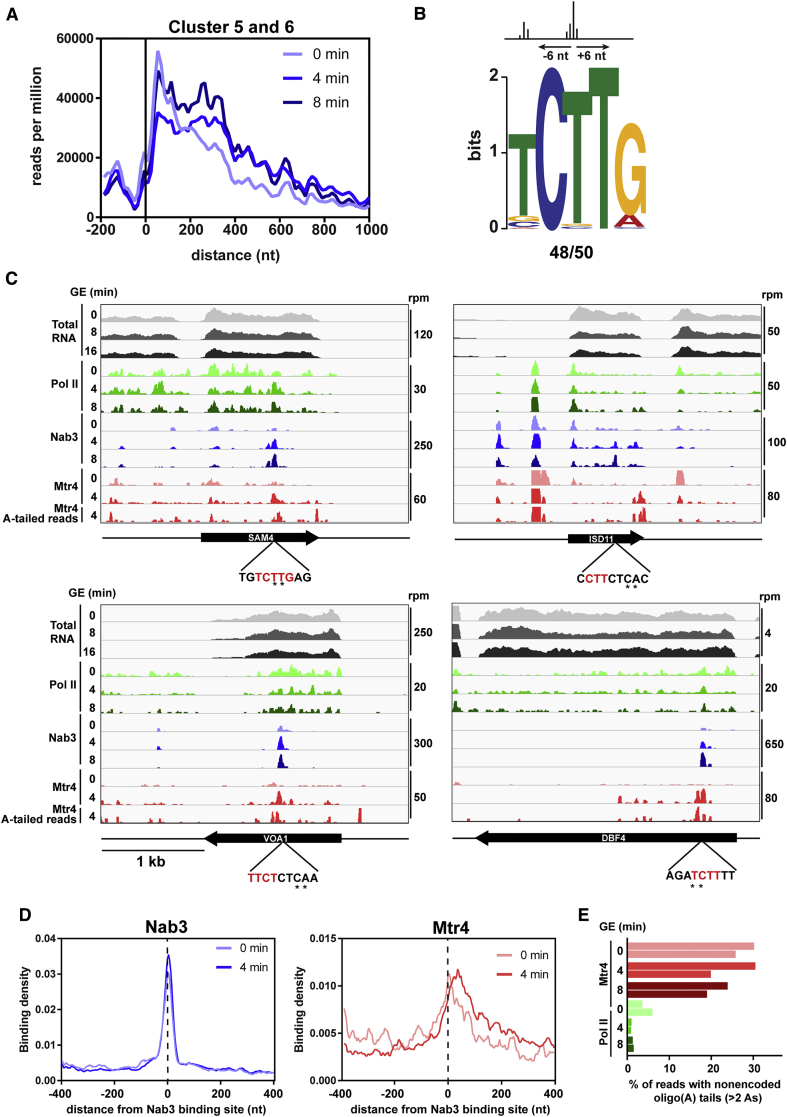
Altered Targeting of NNS and TRAMP during Nutrient Downshift (A) Distribution of reads summed across all cluster 5 and 6 mRNAs between 500 and 2,800 nt in length, aligned by the TSS. (B) Sequence logo of the Nab3 binding site in GE medium, generated by aligning reads ± 6 nt around the top 50 crosslinked sites (defined by sites of microdeletion) among cluster 5 and 6 transcripts, followed by Multiple Em for Motif Elucidation (MEME) analysis. (C) Sequence reads across selected transcripts from cluster 5 and 6. The top three tracks show changes in total RNA levels as determined by RNA-seq. The next eight tracks show the positions of Pol II (green), Nab3 (blue), and Mtr4 (red) across the RNA as determined by CRAC. For Mtr4, the 8-min time point was excluded because of poor sequence coverage. The bottom track shows oligo(A)-tailed reads derived from Mtr4 CRAC at 4 min. The track scales (in reads per million) have been adjusted between different bait proteins and transcripts. The sequence surrounding the primary Nab3 crosslinking site is shown below. The consensus sequence is highlighted in red, and direct crosslinking sites are denoted with asterisks. (D) Relative distribution of Nab3 (left) and Mtr4 (right) across cluster 5 and 6 transcripts. Transcripts were aligned by the strongest site of Nab3 crosslinking within each gene. Each gene was normalized to 1 and weighted equally. (E) Frequency of non-genome-encoded oligo(A) tails in Mtr4 and Pol II CRAC datasets.

The Nrd1-Nab3 heterodimer is recruited to nascent transcripts in part through interactions with phosphorylated Ser5 on the Pol II CTD. This modification is predominant near the transcription start site, potentially explaining why Nrd1 and Nab3 show preferential binding to the 5′ end of protein-coding genes (Schulz et al., 2013, Webb et al., 2014). To test whether NNS is similarly distributed along genes after glucose withdrawal, we aligned all cluster 5 and 6 transcripts by their transcription start site (TSS) and examined Nab3 binding across the meta-transcript. In glucose, Nab3 binding decreased rapidly after the first ∼100 nt. However, after transfer to GE medium, Nab3 occupancy was increased at sites further into the transcript (Figures 3A and S3A). We also normalized genes by transcript length, divided into 50 bins (Figure S3B). This confirmed that mRNAs globally showed relatively less Nab3 binding near the 5′ end in favor of sites further downstream. Nab3 targets (clusters 5 and 6) are similar in size to other mRNAs, with a slight bias toward greater than average length (Figure S3C), and the frequency of CUU Nab3-binding motifs in the TSS proximal regions of cluster 5 and 6 genes was not clearly different from other classes (Figure S3D).

It seemed possible that the altered occupancy of Nab3 reflected changes in binding site specificity. We therefore identified conserved nucleotide sequence motifs surrounding Nab3 binding sites in GE medium. Following UV crosslinking, reverse transcriptase frequently generates errors at the crosslinked nucleotide, leaving characteristic microdeletions in the cDNA sequence (Wlotzka et al., 2011). These were used to identify high-confidence Nab3 binding sites in our datasets. Analysis of the top 50 sites of Nab3 crosslinking in cluster 5 and 6 transcripts on GE medium revealed that 48 conformed to the consensus sequence UCUUG previously defined in glucose medium (Creamer et al., 2011, Wlotzka et al., 2011; Figure 3B). We conclude that the NNS complex recognizes the same consensus motif following nutrient downshift but is now able to bind at sites downstream of the 5′ end.

We also determined the distribution of Pol II, Nab3, and Mtr4 across several individual cluster 5 and 6 transcripts (Figure 3C). Following transfer to GE medium for 8 min, *SAM4*, *ISD11*, *VOA1*, and *DBF4* each showed reduced Pol II occupancy but sharply increased binding to Nab3 and Mtr4. Notably, Mtr4 was frequently bound at or just downstream of the Nab3 binding site (Figure 3C). To examine this more generally, we aligned all cluster 5 and 6 transcripts by the strongest starvation-induced Nab3 binding site within each gene (Figure 3D, left). Following nutrient downshift, Mtr4 was enriched near Nab3 binding sites, with peak binding occurring ∼25 nt downstream (Figure 3D, right). Notably, no enrichment was observed upstream of the Nab3 binding site.

These results suggested that the Nrd1-Nab3 heterodimer recruits Mtr4 (and, presumably, the TRAMP complex) to degrade the RNA. To further test this hypothesis, we analyzed reads associated with Mtr4 for the presence of non-genome-encoded oligo(A) tails (Figure 3E), a characteristic mark of TRAMP-mediated RNA decay. As a control, we additionally analyzed A tails from Pol II CRAC reads. Between 20% and 30% of reads derived from Mtr4 CRAC included oligo(A) tails of at least 2 nt versus 1%–6% for Pol II. The distribution of oligo(A)-tailed reads across *SAM4*, *ISD11*, *VOA1*, and *DBF4* coincided with Mtr4 binding peaks (Figure 3C, bottom track), indicating that Mtr4 recruitment is linked to transcript decay.

### Altered Nab3 Binding Does Not Change Pol II Occupancy

NNS binding has been linked to transcription termination. Hence, it might have been anticipated that Pol II occupancy downstream of the Nab3 binding site would be relatively reduced after glucose withdrawal, particularly on genes from clusters 5 and 6 (Figure 3). However, Pol II occupancy was essentially unaltered following nutrient downshift (Figures S4A, S4B, and S4E). Even genes with notably increased Nab3 binding (e.g., *SAM4*, *ISD11*, *VOA1*, and *DBF4*) showed no evidence of relatively reduced Pol II occupancy downstream of the Nab3 binding site (Figure 3C). In contrast, snoRNAs showed strongly reduced transcription downstream of Nab3 binding sites (Figures S4C and S4D), consistent with previous reports (Steinmetz et al., 2001). These results suggest that NNS recruitment does not always lead to closely positioned transcription termination.

### Ribosomal Protein mRNAs Are Targeted by the TRAMP Complex

**Figure 4:**
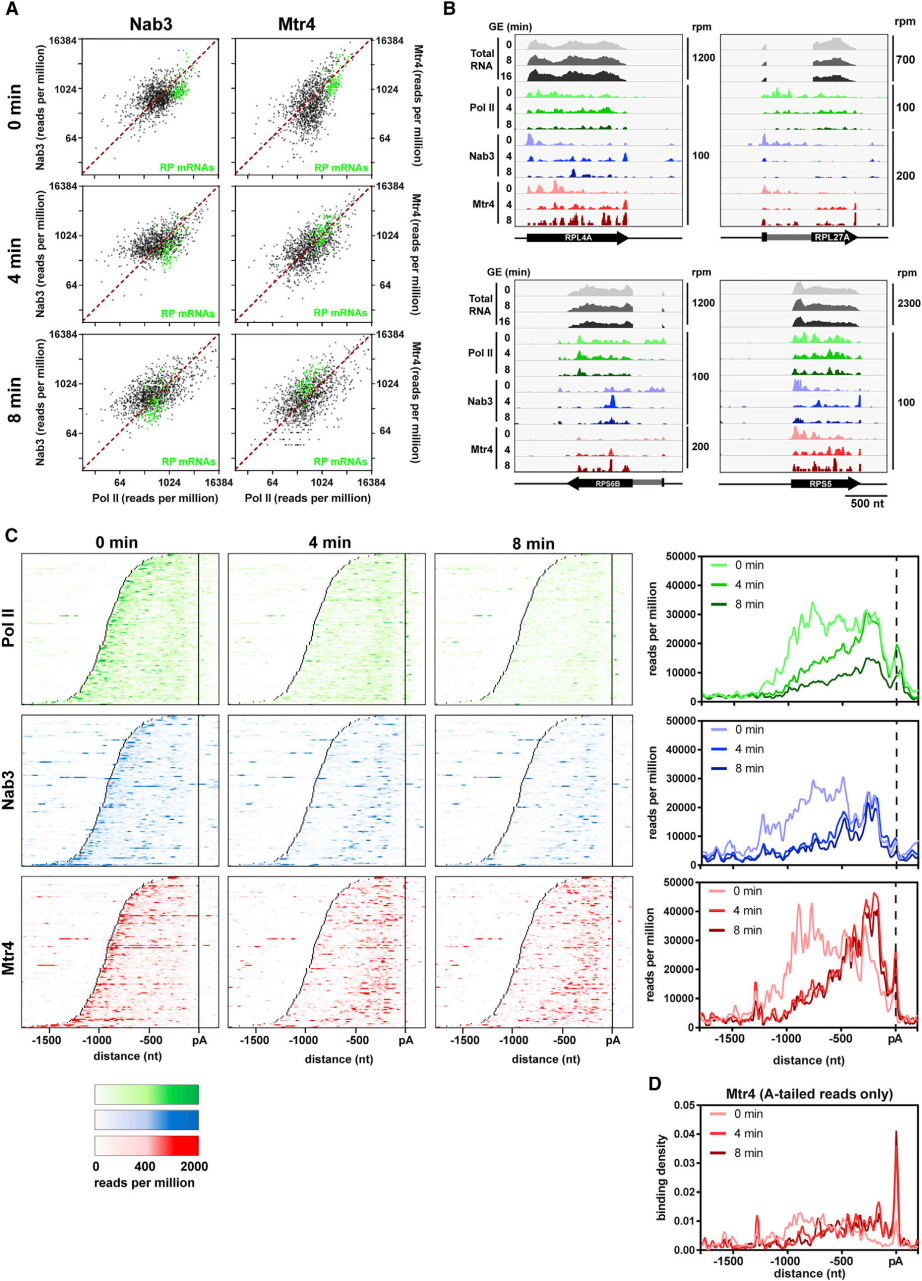
RP mRNAs Are Targeted by TRAMP (A) Nab3 (left) and Mtr4 (right) binding to mRNAs plotted against Pol II binding 0, 4, and 8 min after glucose withdrawal. RP transcripts are highlighted in green. (B) Pol II, Nab3, and Mtr4 binding profiles across selected RP mRNAs. (C) Heatmaps showing the distribution of Pol II, Nab3, and Mtr4 across all RP transcripts. Transcripts were aligned by their poly(A) site and sorted by length from top to bottom. Transcript boundaries are indicated by the two black lines. Summary metagene plots are shown to the right. Heatmaps and metagene plots show the average of two replicates, except Nab3 at 4 min, for which only one replicate was available. (D) Metagene plot of Mtr4 CRAC A-tailed reads across RP transcripts.

Many transcripts, particularly those falling in cluster 7, were strongly targeted by Mtr4 despite reduced overall Pol II association (Figure 2). GO analysis revealed that these genes were highly enriched for functions related to the ribosome or translation. Indeed, 34 of 137 RP mRNAs fall into cluster 7 (Figure 2, highlighted in green). To further investigate this observation, we plotted Pol II versus Mtr4 binding for 1,570 mRNAs and highlighted all ribosomal protein (RP) transcripts in green. RP mRNAs generally showed low levels of Mtr4 binding relative to Pol II in glucose medium (Figure 4A, top right) but greater binding following nutrient downshift (Figure 4A, center and bottom right). In contrast, the Nab3:Pol II ratio for RP mRNAs was mostly unchanged during downshift (Figure 4A, left).

Inspection of the distributions of Nab3 and Mtr4 across individual RP mRNAs (Figures 4B and S5A) showed that Mtr4 binding frequently coincided with Nab3 binding sites. In addition, both proteins accumulated toward the 3′ end of the transcript following downshift. This is in striking contrast to the TSS-proximal binding generally observed in glucose medium. To test whether this is a general feature, we generated heatmaps and metagene plots showing Pol II, Nab3, and Mtr4 binding across all RP transcripts, aligned by the poly(A) site. Pol II binding decreased dramatically with glucose withdrawal (Figure 4C, top), consistent with sharply reduced transcription. Nab3 binding was mostly lost at the 5′ end during downshift, presumably reflecting reduced transcription initiation on these genes, but was maintained or strengthened at distal sites (Figure 4C, center). Mtr4 showed a similar pattern and was also notably enriched at the poly(A) site itself.

**Figure 5:**
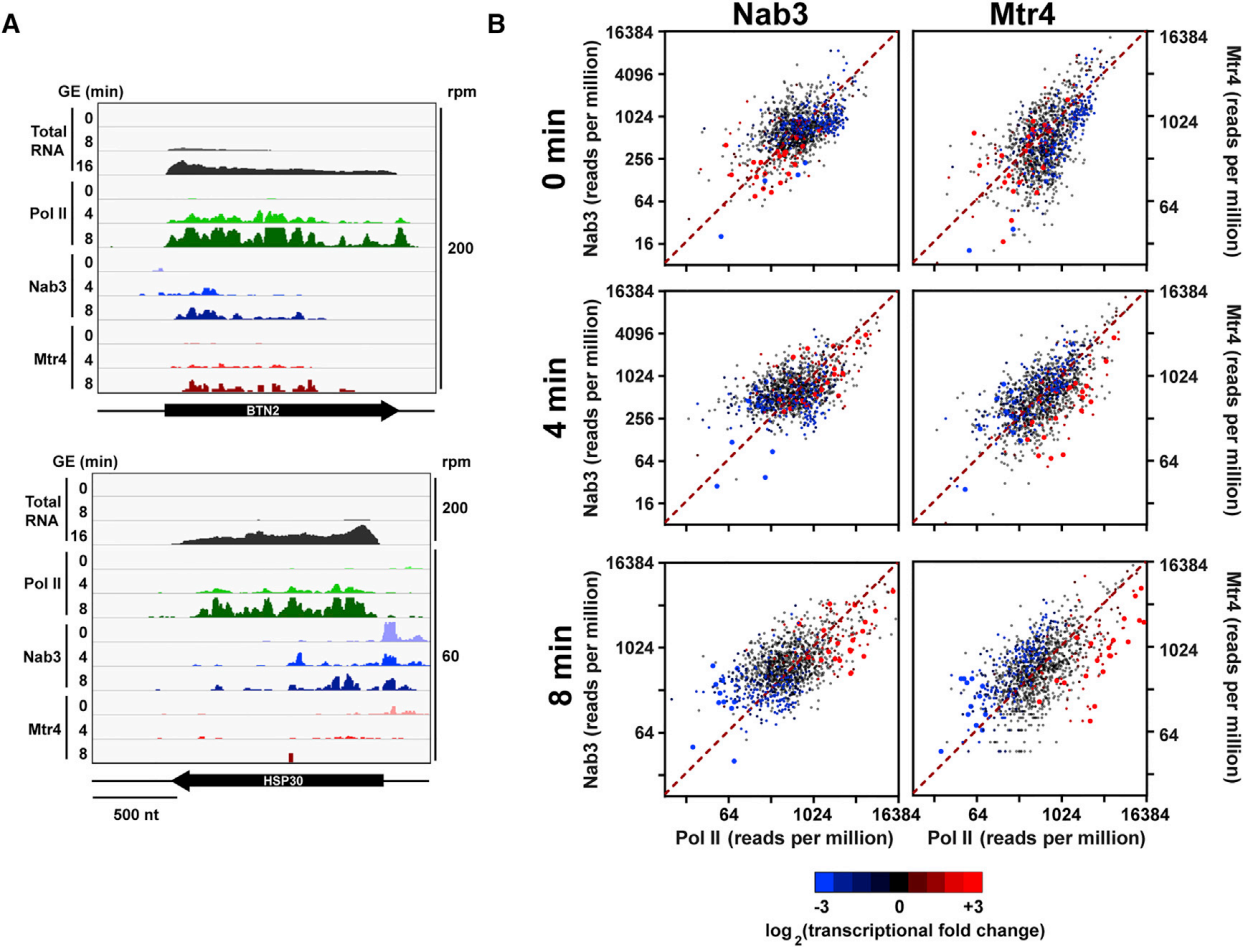
Upregulated Transcripts Are Not Targets of Nab3 and Mtr4 (A) Sequence reads across *BTN2* (top) and *HSP30* (bottom), two of the most transcriptionally upregulated transcripts in our dataset. The top three tracks show changes in total RNA levels. The next nine tracks show the binding profiles of Pol II, Nab3, and Mtr4. (B) Mtr4 (left) or Nab3 (right) binding (y axis) plotted against Pol II binding (x axis) at 0, 4, or 8 min after shift to GE medium. Transcriptionally induced genes are colored red, and transcriptionally downregulated genes are colored blue. A color legend is included below. Genes showing a change of at least ± 3 log_2_ (fold change) are shown as larger dots.

To determine whether Mtr4 binding at the poly(A) site occurred before or after 3′ end processing, we extracted Mtr4 CRAC reads ending with non-genome-encoded A-tracts and plotted these across RP transcripts. A-tailed reads were strongly enriched at the poly(A) site (Figure 4D), suggesting that Mtr4 targets cleaved and polyadenylated RP mRNAs. We next examined oligo(A) tail length for reads mapping to RP transcripts. In glucose-replete medium, oligo(A) tails mapping to the body of the transcript averaged ∼4 nt in length, consistent with TRAMP activity (Figure S5B, left). Oligo(A) tails mapping near the poly(A) site were significantly longer, suggesting that they were generated by canonical polyadenylation. As a positive control, we also examined A tail length from CRAC reads derived from Nab2, the yeast nuclear poly(A) binding protein (Figure S5C). Following downshift, the average oligo(A) tail length associated with Mtr4 tended to increase, but the distinct length increase at the poly(A) site was lost (Figure 5SB, center and right). This could mean that TRAMP-mediated oligoadenylation is more active during glucose starvation or that the decay machinery is saturated. It would also be consistent with formation of the oligo(A) tail by TRAMP at the normal site of polyadenylation.

To determine whether Nab3/Mtr4 bind before or after splicing, we extracted reads mapping across either exon-exon (spliced) or intron-3′ exon (unspliced) splice junctions and calculated the spliced:unspliced ratio for all RP transcripts. In glucose-replete medium, Mtr4 binds unspliced and spliced RP mRNAs in equal proportion (Figure S5D). However, during nutrient downshift, Mtr4 was mainly bound to spliced RP transcripts, consistent with posttranscriptional targeting. In contrast, Nab3 mainly bound unspliced transcripts throughout the time course. This suggests that Nab3 binds cotranscriptionally and is subsequently released before the transcript is degraded. Consistent with this model, strong Nab3 binding was frequently observed at 4 min that was lost by 8 min (Figures 4B, 4C, and S4A). Mtr4, by contrast, remained bound throughout the time course.

### Transcriptionally Induced Genes Escape Nuclear Decay

The results presented so far suggest that NNS and TRAMP are targeted to protein-coding mRNAs as a means of downregulating gene expression. We wondered whether this was generally true or whether some transcripts could evade nuclear decay. Many genes are transcriptionally upregulated during nutrient downshift (Figure 1C), and it would be counterproductive for the cell to immediately degrade them. We initially examined binding profiles for *BTN2* and *HSP30*, two of the most transcriptionally induced genes in our dataset (Figure 5A). Despite dramatic transcriptional induction, both transcripts showed only minor increases in binding of Nab3 or Mtr4. Thus, specific mRNAs escape the generally enhanced binding by NNS and TRAMP following nutrient downshift.

To examine this more generally, we plotted Nab3 or Mtr4 binding against Pol II and colored each mRNA by its transcriptional fold change. Following nutrient shift, highly upregulated genes (red) showed low levels of Nab3 or Mtr4 binding relative to transcription, whereas most highly downregulated genes (blue) showed high levels of binding (Figure 5B). Thus, Nab3 and Mtr4 preferentially target transcripts that are also transcriptionally downregulated in response to glucose withdrawal. Conversely, most transcriptionally induced genes appear to escape targeting by Nab3 and Mtr4.

To gain a mechanistic understanding, we focused on the transcriptionally induced genes *TYE7* and *CTH1*. Tye7 is a transcription factor for the glycolytic gene *ENO1* (Sato et al., 1999), whereas Cth1 is an RNA decay factor that targets genes involved in oxidative phosphorylation (Puig et al., 2008). Both transcripts have extremely high levels of Nab3 binding in glucose-replete medium, but this falls to a “normal” level after nutrient downshift (Figure S6A).

**Figure 6:**
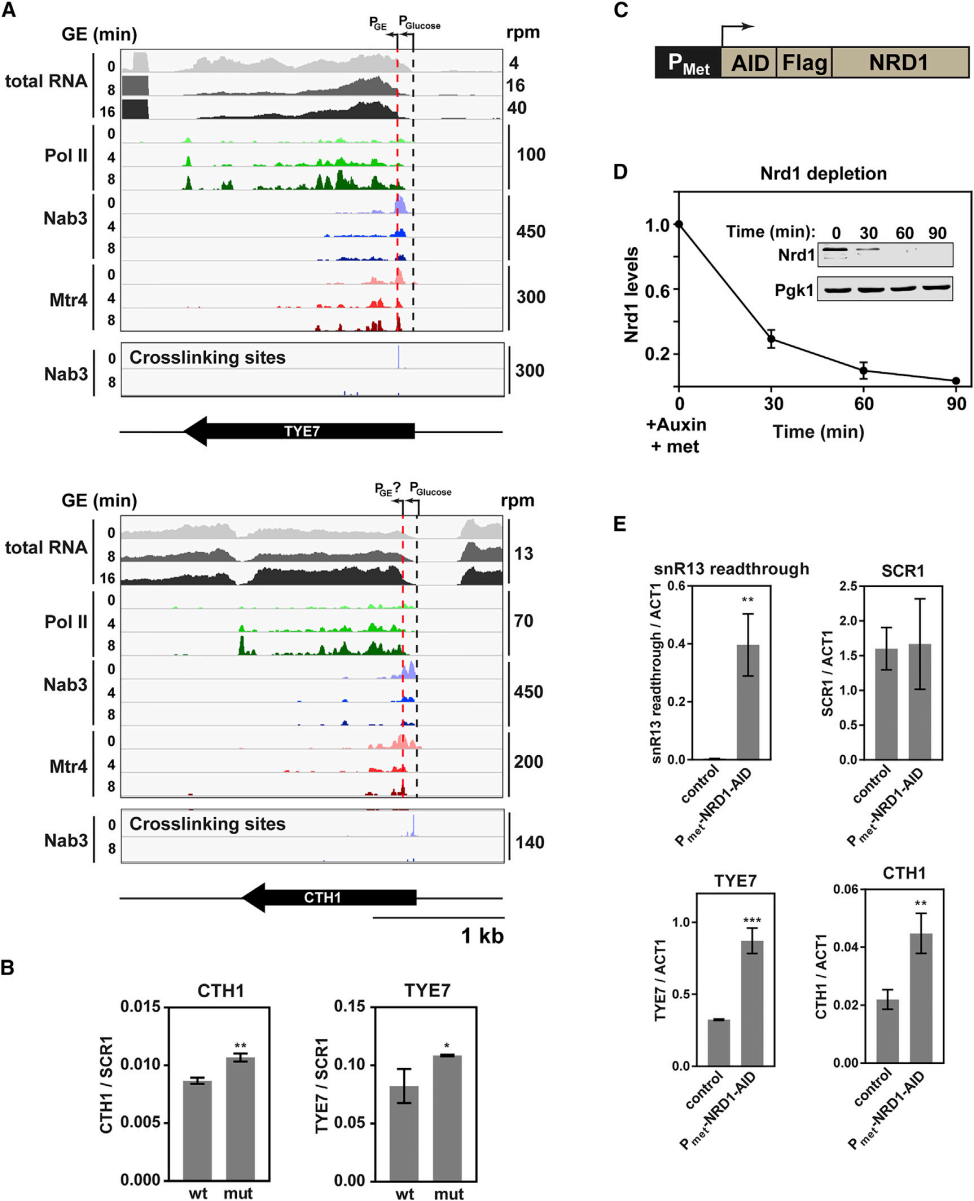
Upregulated Transcripts Show Evidence of Reduced Nuclear Decay (A) Sequence reads across *TYE7* and *CTH1*.The top three tracks show changes in total RNA. Note the different scales. The next nine tracks show the binding profiles of Pol II, Nab3, and Mtr4 across the RNA as determined by CRAC. The bottom two tracks map the deletions (the putative site of crosslinking) from reads derived from Nab3 CRAC. The annotated TSS for both genes is marked by a black dotted line, and the red dotted line marks the putative TSS following nutrient downshift. (B) Bar graph comparing the relative abundance of wild-type (WT) *TYE7* or *CTH1* to mutant (mut) transcripts in which the Nab3 binding site has been removed. Transcript levels were measured by qRT-PCR and normalized to scR1. The values are averages, and error bars are SD (n = 3). (C) Schematic showing the Nrd1 depletion construct. The endogenous *NRD1* gene was placed under control of the inducible P_met_ promoter and tagged with the AID and FLAG. (D) Quantification of Nrd1 protein levels following the addition of auxin and methionine to the medium. Nrd1 protein levels were measured using the anti-FLAG antibody and normalized to Pgk1 levels. Values are averages, and error bars show SD (n = 2). Inset: western blot showing Nrd1 and Pgk1 protein levels for one of the replicates. (E) Quantification of the relative abundance of various transcripts following 90-min Nrd1 depletion relative to a wild-type control. RNA levels were assessed by qRT-PCR, and *ACT1* was used as a loading control. snR13 readthrough was a positive control to assess the effectiveness of Nrd1 depletion, and scR1 was used as a negative control. Values represent averages, and error bars show SD (n = 3).

Inspection of RNA-seq traces revealed that *TYE7* is transcribed from two alternative promoters. *TYE7* transcription predominately initiates at the annotated transcription start site during growth on glucose but from a downstream promoter following nutrient downshift (Figure 6A, compare tracks 1 and 2). Notably, Nab3, and Mtr4 show strong binding between the two transcription start sites, suggesting that transcripts originating from the upstream promoter are subject to surveillance factor binding, potentially leading to premature transcription termination and decay. *CTH1* showed a similar pattern, with transcription initiation during downshift largely occurring downstream of the Nab3 binding site (Figure 6B, compare tracks 3 and 4).

Based on these observations, we hypothesized that NNS activity regulates *TYE7* and *CTH1* transcript levels during growth on glucose. To test this model, we mutated the major Nab3 binding site in each gene, identified from the crosslinking sites (bottom two tracks in Figures 6A and 6B) and assayed RNA levels by qRT-PCR (Figure 6C). Transcripts lacking the Nab3 binding site were upregulated compared with the wild-type control. However, the increase was relatively modest, suggesting that mutation of a single Nab3 target site is insufficient to completely abrogate binding. Thus, we next inactivated the NNS pathway directly by depleting Nrd1. The *NRD1* gene was placed under control of the methionine-repressible *MET25* promoter (Solow et al., 2005) and tagged with the auxin-inducible degron (AID) (Nishimura et al., 2009; Figure 6D). Addition of auxin plus methionine to the medium triggered Nrd1 degradation and blocked the synthesis of new mRNA. This combination resulted in rapid depletion of the Nrd1 protein (∼95% in 90 min; Figure 6D) but did not affect growth (Figure S6B). Following Nrd1 depletion, 3′-extended snR13 strongly accumulated, as previously reported (Tudek et al., 2014, Vasiljeva et al., 2008), and both *TYE7* and *CTH1* mRNAs were significantly upregulated (Figure 6E).

These results indicate that NNS/TRAMP constitutively target *TYE7* and *CTH1* in glucose-replete medium, whereas reduced targeting contributes to transcript upregulation during nutrient downshift. Mechanistically, this is similar to the *FKS2* transcript, whose expression is regulated through the use of alternative promoters on either side of an NNS target site (Kim and Levin, 2011).

## Discussion

Previous studies have examined changes in RNA levels in response to glucose starvation. However, steady-state RNA levels reflect the combination of transcription and decay, making it difficult to determine the precise contributions of each. Here we examined transcription via RNA Pol II association and observed transcriptional changes prior to clear steady-state differences for many genes.

**Figure 7:**
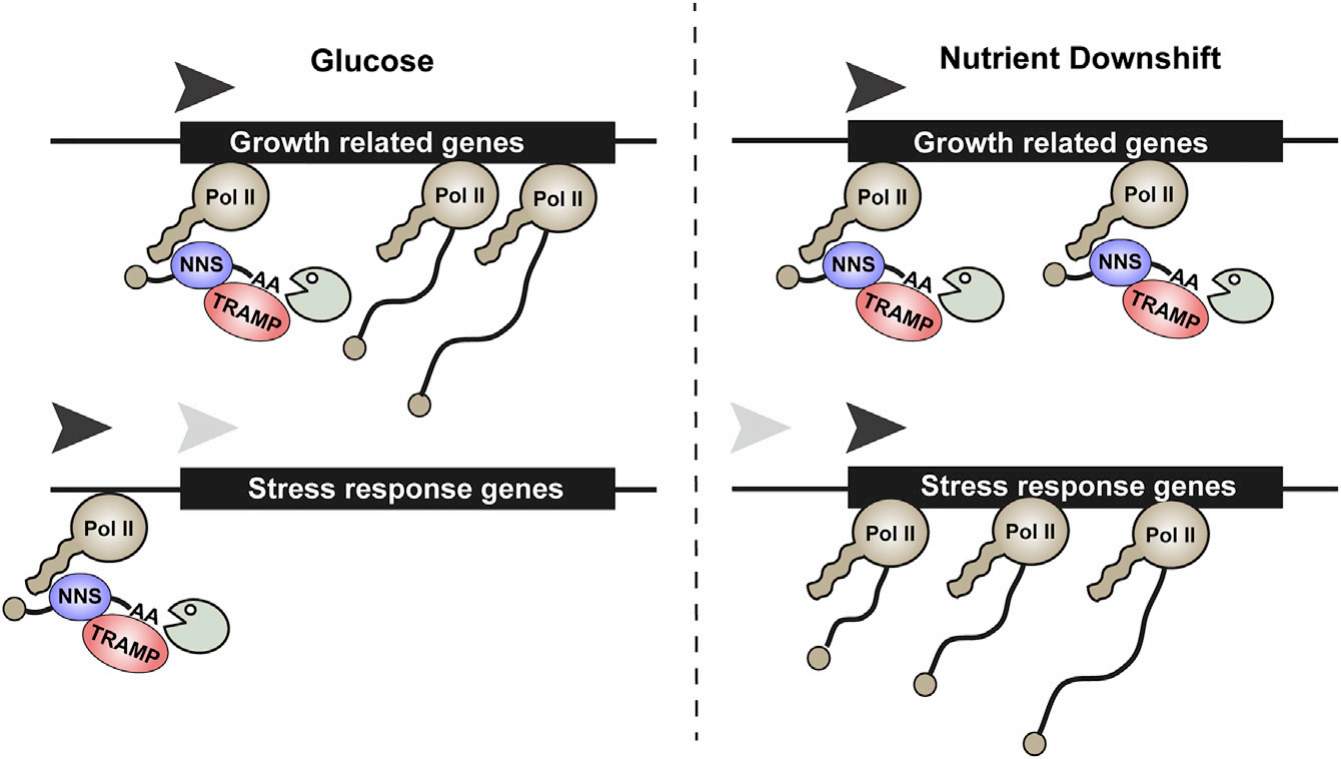
Model for Nuclear Surveillance Activity before and after Glucose Starvation Shown is a diagram depicting the response of Pol II and the nuclear surveillance factors NNS and TRAMP to glucose starvation. During glucose-replete conditions, NNS and TRAMP are mainly restricted to promoter-proximal sites, likely targeting the products of abortive transcription. Following glucose withdrawal, NNS and TRAMP bind at sites further downstream, resulting in transcript oligoadenylation and, presumably, degradation by the nuclear exosome complex (shown as Pac-Man). Stress response genes are transcriptionally induced (increased Pol II binding) following nutrient downshift but generally escape enhanced targeting by the nuclear surveillance machinery.

Our data revealed that many transcripts are selectively targeted by the nuclear RNA decay machinery. Following glucose depletion, Nab3 and Mtr4 are targeted to hundreds of protein coding transcripts. This targeting appears to be selective because GO term analyses revealed enrichment for mRNA targets with functions related to energy usage or cell growth. In contrast, stress response genes, which are highly upregulated during glucose starvation, showed relatively low or even decreased Nab3 and Mtr4 binding during nutrient downshift. Combined, these results indicate that, during glucose withdrawal, NNS and TRAMP predominately target transcripts that are also transcriptionally downregulated (Figure 7).

The most prominent class of transcripts targeted by Nab3 and Mtr4 encode proteins of the 15-subunit V-ATPase complex. V-ATPase hydrolyzes ATP to drive protons across the endoplasmic reticulum, Golgi, or vacuolar membranes. In the absence of glucose, the complex is inactivated and disassembled (Kane, 1995) to conserve ATP because V-ATPase is a major consumer of ATP in cells. Four transcripts encoding components of the complex (*VMA16*, *VMA3*, *VPH1*, and *STV1*) were targeted by both Nab3 and Mtr4, as was *VOA1,* which encodes a factor critical for the assembly of the V-ATPase. Two additional transcripts (*VMA5* and *VMA8*) were targeted by Mtr4 (Figure 2; Table S2). Intriguingly, two of the most upregulated genes in our dataset, *BTN2* and *HSP30*, negatively regulate V-ATPase activity (Chattopadhyay et al., 2000), and both genes escape degradation by NNS/TRAMP (Figure 5). These observations suggest that NNS and TRAMP play an important role in modulating vacuolar ATPase activity.

Another major class of transcripts identified in our analysis was ribosomal protein mRNAs. RP synthesis consumes a considerable portion of the cell’s energy during growth, and RP mRNA transcription is coordinately downregulated in response to nutrient starvation (reviewed in Broach, 2012) (Figure 1). Our results indicate that an additional mechanism degrades RP mRNAs posttranscriptionally because nearly all RP transcripts showed increased binding to Mtr4 during nutrient downshift (Figure 4A). Mtr4 is apparently recruited to RP transcripts by the NNS pathway because it frequently bound to Nab3 target sites following glucose starvation (Figures 4B and 4C). Interestingly, Mtr4 was also strongly targeted to the polyadenylation site (Figures 4C and 4D). This is potentially similar to PABPN1-PAP (Poly(A) Polym) decay (PPD) in mammalian cells, in which Mtr4 is posttranscriptionally recruited to the poly(A) tails of target transcripts (Beaulieu et al., 2012, Bresson et al., 2015, Meola et al., 2016).

Most Nab3 and Mtr4 targets showed only a modest drop in transcript levels following glucose starvation over the short time course analyzed here (Figure 3). The nuclear pool of transcripts is small relative to the cytoplasmic fraction, so changes in nuclear stability caused by increased binding to Nab3 and/or Mtr4 may not immediately be reflected in steady-state RNA levels. However, we cannot exclude the possibility that Nab3 and Mtr4 binding does not always target transcripts for degradation. Nrd1 localizes to nuclear foci following glucose starvation (Darby et al., 2012), suggesting that NNS binding might transiently sequester transcripts. This might be analogous to the sequestration of cytoplasmic mRNAs in stress granules. Specific labeling of newly synthesized transcripts should help resolve these questions.

Previous in vitro and in vivo analyses identified a clear consensus RNA binding site (UCUUG) for Nab3 (Creamer et al., 2011, Wlotzka et al., 2011). Notably, although Nab3 was extensively relocalized during nutritional shift, the consensus binding site motif was unaltered and was recovered at almost all (48 of 50) strong in vivo binding sites analyzed after downshift. The prevalence of consensus Nrd1 and Nab3 binding sites is correlated with ncRNA instability (Arigo et al., 2006a, Arigo et al., 2006b, Conrad et al., 2000, Mayer et al., 2012, Schulz et al., 2013, Steinmetz et al., 2001). However, our data suggest that primary RNA sequence is not a key factor in altered RNA stability during nutritional shift. There are many more Nab3 consensus sites in the transcriptome than are actually bound before or after nutrient shift. This could arise because binding is limited by the presence of pre-mRNA packaging factors at target sites. After nutrient shift, the binding of other factors may change, rendering sites accessible. Alternatively, binding might require additional cofactors, and interactions of Nab3 with these factors may be altered following nutrient shift. This could reflect modification of Nab3 or Nrd1 (e.g., phosphorylation) or changes in other factors (e.g., phosphorylation of the CTD of Pol II).

Strikingly, nearly all genes involved in glycolysis were upregulated during nutrient downshift (Figure 1; Table S4). Most of these genes encode reversible enzymes that catalyze both glycolysis and gluconeogenesis. However, two of the three most upregulated transcripts were *HXK1* and *GLK1*, encoding enzymes that catalyze only the glycolytic reaction. Moreover, two specifically gluconeogenic genes (*FBP1* and *PCK1*) were not expressed before or immediately after glucose depletion. The initial response to dwindling energy reserves may be to metabolize residual glucose by upregulating glycolysis. However, these proteins have all been reported to bind RNA (Beckmann et al., 2015), so we cannot exclude additional functional consequences of their upregulation under conditions in which their catalytic activity is less required.

Several glucose-sensing pathways allow yeast to perceive and respond to changes in glucose levels (reviewed in Conrad et al., 2014). Together, these pathways mediate global changes in both transcription and translation. Previous analyses have shown rapid changes in the behavior of cytoplasmic mRNAs on short timescales following glucose withdrawal, with loss of mRNAs from polysome fractions and relocation to P bodies within 10 min (Ashe et al., 2000, Brengues and Parker, 2007). Retargeting of Nab3 and Mtr4 binding showed similar kinetics following glucose withdrawal, with dramatic changes in Nab3/Mtr4 targeting within 4 min. Such a rapid response suggests that a direct signaling system connects glucose sensing with NNS and TRAMP activity. Consistent with this model, Nrd1 and Nab3 interact genetically with genes involved in the major glucose-sensing pathways, protein kinase A (PKA), and Snf1 signaling (Conrad et al., 2000, Darby et al., 2012), and Nrd1 is dephosphorylated in response to glucose withdrawal (Darby et al., 2012). Understanding how NNS and TRAMP are directed to target transcripts will yield important insights into RNA biology and cellular signaling pathways.

## STAR★Methods

### Key Resources Table

**Table:** 

REAGENT or RESOURCE	SOURCE	IDENTIFIER
**Antibodies**		

Mouse anti-Flag	Sigma	Cat#F3165; RRID: AB_259529
Mouse anti-Pgk1	Thermofisher	Cat#PA528612; RRID: AB_2546088
Goat anti-mouse AlexFluor 700	Invitrogen	Cat#A21036

**Chemicals, Peptides, and Recombinant Proteins**		

-Trp synthetic dropout mix	Formedium	Cat#DCS0149
-Met synthetic dropout mix (Kaiser)	Formedium	Cat#DSCK072C
Methionine	Sigma	Cat#M9625
Guanidine hydrochloride	Sigma	Cat#G4505-1KG
Recombinant TEV protease	Edinburgh Protein Production Facility	N/A

**Critical Commercial Assays**		

cOmplete EDTA-free protease inhibitor cocktail tablets	Roche	Cat#11873580001
Ni-NTA Superflow	QIAGEN	Cat#30410
Pierce spin columns snap cap	Thermo Scientific	Cat#69725
RNace-It Ribonuclease cocktail	Agilent	Cat#400720
TSAP Thermosensitive Alkaline Phosphatase	Promega	Cat#M9910
RNasin Ribonuclease Inhibitor	Promega	Cat#N2115
Recombinant RNasin Ribonuclease Inhibitor	Promega	Cat#N2511
T4 RNA Ligase 1	NEB	Cat#M0204L
T4 PNK	NEB	Cat#M0201L
Nitrocellulose membranes	GE Healthcare	Cat#10 439 196
MetaPhor agarose	Lonza	Cat#50180
NuPAGE 4-12% polyacrylamide Bis-Tris Gels	Life Technologies	Cat#NP0335
NuPAGE LDS 4x sample buffer	Life Technologies	Cat#NP0007
NuPAGE SDS-MOPS running buffer	Life Technologies	Cat#NP0001
NuPAGE Transfer Buffer	Life Technologies	Cat#NP00061
MinElute Gel Extraction kit	QIAGEN	Cat#28604
Proteinase K	Roche	Cat#03115836001
RNase H	NEB	Cat#M0297L
LA Taq	Takara	Cat#RR002M

**Deposited Data**		

Raw data files from CRAC and RNA-seq	NCBI Gene expression omnibus	GEO: GSE86483

**Experimental Models: Organisms/Strains**		

S. cerevisiae Strain background: BY4741 (*MATa his3Δ1 leu2Δ0 met15Δ0 ura3Δ0*)	Longtine et al., 1998	N/A
BY4741 TIR1:his3	David Barrass and Jean Beggs	N/A
ySB036 (BY4741 TIR1:his3 MET15)	This paper	N/A
ySB043 (BY4741 TIR1:his3 MET15 *P*_*MET25*_*-*AID-NRD1)	This paper	N/A

**Recombinant DNA**		

pFA6a-HIS3-MX6	Longtine et al., 1998	N/A
pSB031 (Hyg+-*P*_*MET25*_*-*Kozak-start codon-AID^∗^-6xFlag)	This study	N/A

**Sequence-Based Reagents**		

A full list of DNA oligos is presented in Figure S5.		N/A

**Software and Algorithms**		

PyCRAC	Webb et al., 2014	https://bitbucket.org/sgrann/pycrac
HISAT2 v2.02	Kim et al., 2015	https://ccb.jhu.edu/software/hisat2/index.shtml
SAMtools v1.3.1		http://www.htslib.org/
Bedtools v2.25		https://github.com/arq5x/bedtools2
featureCounts v1.4.2		http://bioinf.wehi.edu.au/featureCounts/
MEME		http://meme-suite.org/
Prism 7	Graphpad	http://www.graphpad.com
Integrative Genomics Viewer	Broad Institute	http://software.broadinstitute.org/software/igv/

### Contact for Reagent and Resource Sharing

Further information and requests for reagents may be directed to and will be fulfilled by the corresponding author, Dr. David Tollervey (d.tollervey@ed.ac.uk).

### Method Details

#### Strains

All *S. cerevisiae* strains are derived from BY4741 (*MATa his3Δ1 leu2Δ0 met15Δ0 ura3Δ0*). For CRAC, bait proteins were C-terminally tagged with HTP (His_6_-TEV cleavage site- 2X Protein A). The Nab3-HTP (Wlotzka et al., 2011), Mtr4-HTP (Tuck and Tollervey, 2013), and Rpo21-HTP (Milligan et al., 2016) strains were previously described.

#### Growth conditions

For UV crosslinking, strains were grown overnight in –TRP synthetic dropout medium with 2% glucose, diluted to OD_600_ 0.05 in 3L media and grown to OD_600_ 0.5 at 30°C. Cells were collected by filtration, washed with –TRP medium containing 2% glycerol and 2% ethanol (GE), and transferred to fresh –TRP medium containing GE. Cells were then grown for an additional 4 or 8 min before UV crosslinking. As a control, a second culture was grown in parallel and crosslinked while still in glucose containing medium (‘0’ time point), or filtered and transferred to fresh glucose-containing medium (‘mock’).

Addition of auxin (Indole-3-acetic acid from Alfa Aesar) impaired cell growth in standard SD media but not in Kaiser SD, which was therefore used for Nrd1 depletion experiments. Cells were grown overnight in 2% glucose medium lacking methionine, diluted to OD_600_ 0.05 in 1 L culture and grown to OD_600_ 0.3 at 30°C. Auxin and methionine were added to final concentrations of 1 mM and 670 μM, respectively, and cells were cultured for an additional 90 min (OD_600_ ∼0.5) prior to harvesting.

#### Crosslinking and Analysis of cDNAs (CRAC)

The CRAC protocol used here was performed basically as previously described (Granneman et al., 2011, Tuck and Tollervey, 2013).

Approximately 3 L of cells at OD_600_ 0.5 were UV-irradiated at 254 nm for 100 s using the Megatron (Granneman et al., 2011). Cells were split into three 1 L aliquots and centrifuged for 12 min at 2700 x g, resuspended in 30 mL ice-cold PBS, and centrifuged at 4600 x g for 20 min. The resulting pellets were stored at −80°C.

Cell pellets (derived from approximately 1 L of 0.5 cell culture) were vortexed with 1 mL TN150 (50 mM Tris-HCl pH = 7.8, 150 mM NaCl, 0.1% NP-40, 5 mM β-mercaptoethanol, and EDTA-free protease inhibitor cocktail (1 tablet per 50 mL)) and 2.5 mL zirconia beads (Thistle Scientific) for 5x 1min pulses, and cooled on ice in between. Cell lysates were diluted with an additional 3 mL TN150, briefly vortexed, and centrifuged at 4600 x g for 20 min. The supernatant was moved to a fresh tube and spun for an additional 20 min at 16000 x g. Cleared lysates were incubated with 125 μL IgG beads (IgG Sepharose 6 Fast Flow), nutating at 4°C for 2 hr. Beads were washed with TN1000 (2 × 10 mL for 5 min; same as TN150, but with 1 M NaCl), and TN150 (2 × 10 mL). Subsequently, the beads were incubated with 600 μL TN150 and 3 μL homemade GST-TEV (2 hr, 18°C with shaking) in order to elute His-tagged protein:RNA complexes. The eluate was collected by passing through a spin column (Pierce) and then treated with RNace-IT (Agilent; 0.1 units, 5 min, 37°C) to fragment protein-bound RNA. The RNase reaction was quenched with the addition to 400 mg guanidine hydrochloride. The solution was adjusted for nickel affinity purification with the addition of 27 μL NaCl (5.0 M) and 3 μL imidazole (2.5 M), and added to 50 μl washed nickel beads (Ni-NTA agarose, QIAGEN).

Following an overnight incubation, the nickel beads were transferred to a spin column and washed three times with WBI (6.0 M guanidine hydrochloride, 50 mM Tris-HCl pH = 7.8, 300 mM NaCl, 0.1% NP-40, 10 mM imidazole, and 5 mM β-mercaptoethanol), and then three times with 1xPNK buffer (50mM Tris-HCl pH = 7.8, 10 mM MgCl_2_, 0.5% NP-40, and 5 mM β-mercaptoethanol). Subsequent reactions (80 μL total volume for each) were performed in the columns, and afterward washed once with WBI and thrice with 1xPNK buffer:

1. Phosphatase treatment (1x PNK buffer, 8 u TSAP (Promega), 80 u RNasIN (Promega); 37°C for 30 min).
2. 3′ linker ligation (1x PNK buffer, 40 u T4 RNA ligase I (NEB), 80 u RNasIN, 1 μM preadenylated 3′ miRCat-33 linker (IDT); 25°C for 6 hr).
3. 5′ end phosphorylation and radiolabeling (1x PNK buffer, 40 u T4 PNK (NEB), 40μCi ^32^P-γATP; 37°C for 60 min, with addition of 100 nmol of ATP after 40 min).
4. 5′ linker ligation (1x PNK buffer, 40 u T4 RNA ligase I (NEB), 80 u RNasIN, linker, 1 mM ATP; 16°C overnight).

The beads were washed three times with WBII (50 mM Tris-HCl pH = 7.8, 50 mM NaCl, 0.1% NP-40, 10 mM imidazole, and 5 mM β-mercaptoethanol). Protein:RNA complexes were eluted for 2x10 min in 125 μL elution buffer (same as WBII but with 150 mM imidazole), added to 1.25 mL acetone, and precipitated for at least two hours at −20°C. RNPs were pelleted by centrifugation at 16000 g for 20 min, and resuspended in 20 μL 1x NuPAGE LDS sample loading buffer (Life Technologies). After electrophoresis (4%–12% Bis-tris NuPAGE gel, Invitrogen; 150 V), protein:RNA complexes were transferred to Hybond-C nitrocellulose membranes (Amersham) (1x NuPAGE transfer buffer (Invitrogen); 1.5 hr, 100 V). Labeled RNA was detected by autoradiography. The appropriate regions were excised from the membrane and treated with 0.25 μg/μL Proteinease K (50 mM Tris-HCl pH = 7.8, 50 mM NaCl, 0.1% NP-40, 10 mM imidazole, 1% SDS, 5 mM EDTA, and 5 mM β-mercaptoethanol; 2 hr 55°C with shaking). RNA was isolated with phenol:chloroform extraction followed by ethanol precipitation.

RNA was reverse transcribed using Superscript III (Life Technologies) and the miRCat-33 RT oligo (IDT) for 1 hr at 50°C in a 20μL reaction. Samples were heat inactivated (65°C, 15 min) and then treated with RNase H (NEB; 37°C, 30 min). cDNA was amplified by PCR in three separate reactions using LA Taq (Takara; 4μL template, 18-24 cycles, 52° annealing). PCR reactions were combined and precipitated in ethanol, and resolved on a 3% Metaphore agarose gel. A region corresponding to ∼130 to 250 nt was excised from the gel and extracted using the Gel extraction min-elute kit (QIAGEN). Libraries were then submitted for Illumina Hiseq-2000 at Edinburgh Genomics.

#### RNA sequencing

Samples were rRNA depleted using the Ribo Zero yeast kit (Epicenter) and sequencing libraries were prepared using the Truseq Stranded Total RNA sequencing kit (Illumina), as recommended by the manufacturers. The RiboZero (yeast) kit was substituted in the protocol for the Ribozero kit supplied with the Truseq Stranded Total RNA sequencing kit. Sequencing of the libraries was carried out on an Illumina HiSeq 2500 in Rapid output mode using the Illumina Truseq Rapid v1 chemistry.

#### Bioinformatic analysis

##### Sequencing analysis

Raw sequencing data were preprocessed using the fastx toolkit as previously described (Tuck and Tollervey, 2013). Preprocessing included fastx_clipper to remove 3′ sequencing adapters, fastq_quality_trimmer to remove low quality bases from the ends of reads, fastq_quality_filter to remove low quality reads, and fastx_artifacts_filter to remove homopolymeric sequencing artifacts.

The 5′ linkers used to generate CRAC libraries contained three random nucleotides, enabling identical reads putatively arising from PCR amplification of a single cDNA to be collapsed into a single read. The 5′ linkers also included a barcode, allowing multiple samples to be sequenced simultaneously. Reads with different barcodes were subsequently demultiplexed with the pyBarcodeFilter command in the pyCRAC software (Webb et al., 2014).

Processed reads were then mapped to the *S. cerevisiae* genome (SGD v64) using Novoalign (Novocraft) and a genome annotation from Ensembl (EF4.68) modified to include UTR sequences and noncoding RNAs. Reads aligning to more than one location in the genome were randomly assigned. Reads mapping to each transcript were counted using pyReadCounters from pyCRAC. For most analyses, we excluded ORFs of unknown function as these frequently correspond to shorter noncoding RNAs rather than full length mRNAs. To examine the distribution of hits across individual genes, we used the pyGTF2bedgraph command in the pyCRAC package to generate bedgraph files. These files were visualized using the Integrated Genomics Viewer.

RNaseq datasets were aligned with HISAT2 (Kim et al., 2015) using a genome annotation from Ensembl (EF4.68). The resulting .sam files were converted to .bam and sorted by chromosome position using the samtools package. Bedgraph files were generated using bedtools genomecov. Finally, reads mapping to each transcript were counted using featureCounts.

##### Metagene plots

To generate metagene plots, we aligned transcripts by various features including transcription start site, poly(A) site, or the Nab3 binding site. This was done by modifying the start and end points of features in the Ensembl genome annotation. In the case of ribosomal protein transcripts, we observed that both the 5′ and 3′ ends were frequently misannotated. Thus, we manually assigned these using data from RNaseq, Cbc1 CRAC (to mark the 5′ end), and Nab2 CRAC (to mark the 3′ end). For snoRNAs, the transcribed unit typically extends beyond the mature 5′ and 3′ ends which are annotated in Ensembl. Thus, for the metagene plots in Figures S4C and S4D, we manually determined the snoRNA transcriptional units by visual inspection of Pol II CRAC data.

We then used pyBinCollector from pyCRAC to divide these modified features into equal bins and sum or average the number of reads in each bin. Because all of our metagene plots extended at least 500 nt across the gene, we excluded transcripts shorter than this. For the panels in Figures 3A and 4, we plotted hits per million across the metagene. Thus, more highly bound genes are weighted more heavily. In Figures 3D and S4, the metatranscripts show the average distribution across individual transcripts. For the plot in Figure S3B, we normalized protein coding genes by length, and divided hits into 50 bins across the metagene.

##### Heatmaps and plots

Heatmaps were generated in Excel. The 2D plots (e.g., Figure 1F) were made with Prism 7 (Graphpad). Only genes included in the 2D plots were used when calculating reads per million.

##### Motif analysis

To identify the Nab3 consensus binding motif (Figure 3B), we examined the top 50 crosslinked sites among transcripts from Clusters 2 and 8. Sequences of 13 nt surrounding the crosslinking site were extracted and analyzed using MEME (http://meme-suite.org), set to identify zero or one motif of 3 to 8 nt per transcript. The distribution of CTT motifs across mRNAs (Figure 3D), was assessed using FIMO in the MEME suite.

##### Oligo(A) tail analysis

To identify reads containing non-genome-encoded oligo(A) tails, we used a program developed by Grzegorz Kudla (Tuck and Tollervey, 2013, Wlotzka et al., 2011). We extracted reads containing the 3′ adaptor, and removed the adaptor sequence using the fastx toolkit described above. We then used blastall to align reads to the yeast genome. For some reads, the mapped region did not extend to the 3′ end of the clipped read, and the unmapped nucleotides were considered to be non-genome-encoded. Reads where the non-genome-encoded portion consisted of two or more As (and no more than one in five non-A residues) were classified as non-genome-encoded oligo(A) tails. These reads were then mapped to the genome using Novoalign in order to visualize the distribution of oligo(A) tailed-reads across particular transcripts. For the data in Figures S5B and S5C, we averaged the non-genome-encoded oligo(A)-tail length for RP transcripts at each nucleotide along the RP metagene.

#### Strain construction

Point mutants in *TYE7* and *CTH1* were generated with mismatched primers containing the desired mutation (oligos B and C, see Table S5) and flanking primers (oligos A and D) using SOEing PCR. For both genes, we mutated the Nab3 consensus sequence TCTT to AAAA. The second round of SOEing PCR used flanking primers (oligos A and D) containing PmeI and EcoRI sites. In addition, a wild-type control was generated by using just the flanking primers. In order to leave the endogenous promoter intact, we included sequence upstream of each open reading frame (−531 for TYE7 and −311 for CTH1). Following digestion with PmeI and EcoRI, we cloned the individual fragments separately into pFA6a-HIS3-MX6 (Longtine et al., 1998) cut with the same restriction enzymes. Subsequently, we amplified the *HIS3* allele and the mutated or wild-type *TYE7* and *CTH1* fragments using oligos E and D. The resulting PCR fragments were transformed into yeast and transformants were selected on –HIS SD (synthetic dropout) plates. Clones were verified by genomic DNA extraction and sequencing of the PCR fragment generated by oligos A and F.

#### Expression of the AID-Nrd1 fusion under the control of the P_MET25_ promoter

The auxin inducible degron (AID) system was originally developed by the Kanemaki lab (Nishimura et al., 2009). The addition of auxin to cells activates the E3 ligase Tir1 to target AID-containing proteins for degradation by the proteasome. We used a modified version of the AID referred to as AID^∗^, and a yeast strain with *TIR1* integrated at the *HIS3* locus in BY4741. Both reagents were kindly provided by the laboratory of Jean Beggs (D. Barrass, B. Terlouw, and J. Beggs, personal communication). A BY4741 *TIR1* strain that can grow in the absence of methionine was generated by reintroducing the wild-type *MET15* gene into its endogenous locus. The *MET15* locus was amplified using primers oSB170 and 171 from W303 genomic DNA. The resulting fragment was transformed into yeast and positive clones were selected on SD –Met plates. Clones were verified by genomic DNA extraction and PCR using oSB170 and 171. The resulting strain was named ySB036. The sequence from −382 to −1 upstream of the *MET25* ORF was amplified using W303 genomic DNA with forward and reverse primers (oSB182 and 183) containing HindIII and KpnI restriction sites, respectively. In addition, the reverse oligo contained a consensus Kozak sequence and a start codon just upstream of the restriction site. Following digestion, the *MET25* promoter was cloned into pAID^∗^ digested with the same restriction enzymes. Next, we amplified the Hygromycin resistance gene (Hyg+) from pAID^∗^ using forward and reverse primers (oSB184 and 185) containing PvuII and HindIII restriction sites, respectively. After digestion with PvuII and HindIII, Hyg+ was inserted upstream of *P*_*MET25*_. The resulting plasmid (pSB031) contained, in order: Hyg+-*P*_*MET25*_*-*Kozak-start codon-AID^∗^-6xFlag. We amplified this region using primers (oSB186 and 187) with overhangs matching the upstream sequence of the *NRD1* ORF to facilitate N-terminal insertion by homologous recombination. Because the AID^∗^-flag sequence was originally designed for C-terminal tagging, the plasmid contained a stop codon immediately following the Flag tag sequence. We designed oSB187 to contain a mismatch at this site to convert the stop codon (TGA) to cysteine (TGT). The resulting cDNA was transformed into ySB036 to generate ySB043. The parent strain ySB036 served as a positive control for the experiments in Figures 6 and S6.

#### RT-qPCR

Approximately 10 OD_600_ units of cells were collected by brief centrifugation (4600 rpm; 1 min), resuspended in 1mL ice cold PBS, and centrifuged again to pellet. Total RNA (2 μg) was isolated by the GTC-phenol method and residual DNA was degraded by treating with Turbo DNase (Ambion). DNase was removed by phenol-chloroform-isoamyl alcohol extraction followed by ethanol precipitation. cDNA was synthesized with MuLV reverse transcriptase (Invitrogen) using 200 ng random hexamers (Thermo Scientific). Expression levels of individual transcripts were determined by quantitative PCR using SYBR Green (Clonetech) for detection. cDNA dilutions of 1:10 were used for qPCR. Each sample was measured in technical quadruplicates and the results were averaged together. Relative expression was calculated using ΔΔC_t_ and by normalizing to levels of *ACT1* or *SCR1* in each sample.

#### Western blotting

Protein extracts were loaded onto 10% polyacrylamide-SDS gels and transferred to nitrocellulose membranes. Following blocking in 5% milk, the membrane was incubated overnight in anti-Flag (Sigma F3165) diluted 1:2000, followed by an anti-mouse secondary (Invitrogen A21036) diluted 1:5000. Proteins were visualized using the Li-Cor Odyssey. As a loading control, membranes were subsequently probed using anti-Pgk1 (Thermofisher PA528612) at 1:5000 followed by the same secondary antibody as above.

### Quantification and Statistical Analysis

To assess the reproducibility between datasets (Figure 1B), we calculated the pairwise Pearson’s correlation coefficients between all datasets using Excel. GO term analyses and calculations of statistical significance were performed using the Gene Ontology Consortium website (http://www.geneontology.org). The statistical significance of RT-qPCR results from Figure 6 was assessed using the unpaired Student’s t test.

### Data and Software Availability

The accession number for the sequencing data reported in this paper is GEO: GSE86483.

## Author Contributions

A.T. and D.T. conceived the study. S.B. and A.T. performed most experiments and analyzed the results together with D.T. D.S. generated and analyzed the TYE7 and CTH1 mutant strains. S.B. and D.T. wrote the manuscript.

## Supplemental Information

Document S1. Figures S1–S6

Table S1. The Raw Data Used to Generate Figure 1C, Related to Figure 1

Table S2. The Raw Data Used to Generate Figure 2, Related to Figure 2

Table S3. GO Analysis of Clusters from Figure 2, Related to Figure 2 This table includes GO terms, fold enrichment, and p values for the clusters in Figure 2.

Table S4. Analysis of Glycolytic Genes, Related to Figure 1 This table includes raw data showing the changes in transcription and total RNA for glycolytic genes.

Table S5. Primer Table, Related to STAR Methods This table lists names and sequences of oligos used in this study.

Document S2. Article plus Supplemental Information
