# Supplementary material for: Nuclear RNA Decay Pathways Aid Rapid Remodeling of Gene Expression in Yeast
Source: Mol Cell. 2017 Mar 2;65(5):787–800.e5. doi: 10.1016/j.molcel.2017.01.005 (PMC5344683; doi:10.1016/j.molcel.2017.01.005)
Supplement: Document S1. Figures S1–S6 [file mmc1.pdf]

Molecular Cell, Volume *65*

Supplemental Information

Nuclear RNA Decay Pathways Aid Rapid Remodeling of Gene Expression in Yeast

Stefan Bresson, Alex Tuck, Desislava Staneva, and David Tollervey

SUPPLEMENTAL INFORMATION

SUPPLEMENTAL FIGURE LEGENDS

Figure S1. Related to Figure 1.

(A) Growth curves of a wild type control (BY4741) compared to Rpo21-HTP, Nab3-HTP, and Mtr4-HTP strains. Cells were grow overnight in SD –TRP, diluted to 0.05 OD_{600}, and grown for 7 h. (B) Comparison of Pol II, Nab3, and Mtr4 binding across different functional classes of genes. The average of two independent replicates is shown, except for Nab3 4 min, for which only one replicate was available. (C) Boxplots showing the number of Nab3 CRAC reads per million of all CUTs, SUTs, and mRNAs, before ('+') or 8 min after glucose withdrawal ('-'). The boxes show the 25th, 50th, and 75th percentiles, and whiskers show the 10th and 90th percentiles.

Figure S2. Related to Figure 2.

Venn diagram showing the top 2500 binders to Pol II (green), Nab3 (blue), and Mtr4 (red). The 1570 genes in common were analyzed in Figure 2.

Figure S3. Related to Figure 3.

(A) The distribution of Nab3 across Cluster 5 and 6 mRNAs between 500 and 2800 nt in length. Transcripts are aligned by the transcription start site (TSS) and arranged by length from top to bottom. The black lines show transcript boundaries. (B) Nab3 distribution across all mRNAs. Transcripts were normalized by length and divided into 50 equally sized bins. The average fraction of reads falling in each bin is plotted along the y-axis. (C) Frequency distribution of transcript lengths for Cluster 5 and 6 mRNAs (grey bars) compared to all other mRNAs included in the clustering (black bars). Transcript lengths were grouped into 500 nt bins. (D) Frequency of Nab3-binding motifs in the TSS proximal regions of cluster 5 and 6 genes (grey bars) compared to all other classes (black bars).

Figure S4. Related to Figure 3.

(A) Binding density of Nab3 (*top*) and Pol II (*bottom*) across Cluster 5 and 6 mRNAs. Meta-transcripts were aligned by the strongest site of Nab3 crosslinking within each gene. (B)

Heatmap representations of the data from (A). (C) Binding density of Nab3 (*top*) and Pol II (*bottom*) across snoRNAs. Meta-transcripts were aligned by the strongest site of Nab3 crosslinking within each snoRNAs transcriptional unit. snoRNAs with nearby transcriptional units (27 of 77) were excluded from the analysis. (D) Heatmap representations of the data from (C). (E) Pol II binding density across Cluster 5 and 6 mRNAs. Transcripts were aligned by their transcription start site (TSS).

Figure S5. Related to Figure 4.

(A) Total RNA (grey tracks), and Pol II (green tracks), Nab3 (blue tracks) and Mtr4 (red tracks) binding profiles across additional selected RP mRNAs. (B) Average length of non-templated A-tails associated with Mtr4 reads across RP transcripts. (C) Same as (B) but using reads derived from Nab2 CRAC. Nab2 was analyzed in both a wild type strain and *Δrrp6*, which was previously reported to stabilize the binding of Nab2 to poly(A) tails. (D) Splicing analysis of RP transcripts. The spliced:unspliced ratio was calculated by dividing the number of reads mapping across exon-exon splice junctions by the number of reads spanning the intron-3′ exon splice site.

Figure S6. Related to Figure 5.

(A) The plots from Figure 5B are reproduced with *TYE7* and *CTH1* highlighted in pink and orange, respectively. (B) Growth curve comparing a control strain (OsTIR) to a Nrd1 depletion strain (OsTIR + *NRD1-AID*). The strains continue to grow at the same rate for 90 min following addition of auxin and methionine to the media to deplete Nrd1.

Table S1. Related to Figure 1.

This spreadsheet includes the raw data used to generate Figure 1C.

Table S2. Related to Figure 2.

This spreadsheet includes the raw data used to generate Figure 2.

Table S3. GO analysis of Clusters from Figure 2.

This table includes GO terms, fold enrichment, and p-values for the clusters in Figure 2.

Table S4. Analysis of glycolytic genes. Related to Figure 1.

This table includes raw data showing the changes in transcription and total RNA for glycolytic genes.

Table S5. Primer table. Related to the STAR Methods.

This table lists names and sequences of oligos used in this study.

KEY RESOURCES TABLE

REAGENT or RESOURCE	SOURCE	IDENTIFIER
Antibodies		
Mouse anti-Flag	Sigma	Cat#F3165
Mouse anti-Pgk1	Thermofisher	Cat#PA528612
Goat anti-mouse AlexFluor 700	Invitrogen	Cat#A21036
Chemicals, Peptides, and Recombinant Proteins		
-Trp synthetic dropout mix	Formedium	Cat#DCS0149
-Met synthetic dropout mix (Kaiser)	Formedium	Cat#DSCK072C
Methionine	Sigma	Cat#M9625
Guanidine hydrochloride	Sigma	Cat#G4505-1KG
Recombinant TEV protease	Edinburgh Protein Production Facility	
Critical Commercial Assays		
cOmplete EDTA-free protease inhibitor cocktail tablets	Roche	Cat#11873580001
Ni-NTA Superflow	Qiagen	Cat#30410
Pierce spin columns snap cap	Thermo Scientific	Cat#69725
RNace-It Ribonuclease cocktail	Agilent	Cat#400720
TSAP Thermosensitive Alkaline Phosphatase	Promega	Cat#M9910
RNasin Ribonuclease Inhibitor	Promega	Cat#N2115
Recombinant RNasin Ribonuclease Inhibitor	Promega	Cat#N2511
T4 RNA Ligase 1	NEB	Cat#M0204L
T4 PNK	NEB	Cat#M0201L
Nitrocellulose membranes	GE Healthcare	Cat#10 439 196
MetaPhor agarose	Lonza	Cat#50180
NuPAGE 4-12% polyacrylamide Bis-Tris Gels	Life Technologies	Cat#NP0335
NuPAGE LDS 4x sample buffer	Life Technologies	Cat#NP0007
NuPAGE SDS-MOPS running buffer	Life Technologies	Cat#NP0001

NuPAGE Transfer Buffer	Life Technologies	Cat#NP00061
MinElute Gel Extraction kit	QIAGEN	Cat#28604
Proteinase K	Roche	Cat#03115836001
RNase H	NEB	Cat#M0297L
LA Taq	Takara	Cat#RR002M
Deposited Data		
Raw data files from CRAC and RNAseq	NCBI Gene expression omnibus	GEO: GSE86483
Experimental Models: Organisms/Strains		
S. cerevisiae Strain background: BY4741 (*MATa his3Δ1 leu2Δ0 met15Δ0 ura3Δ0*)	Longtine et al., 1998	
BY4741 TIR1:his3	David Barrass and Jean Beggs	
ySB036 (BY4741 TIR1:his3 MET15)	This paper	
ySB043 (BY4741 TIR1:his3 MET15 P_{MET25}-AID-NRD1)	This paper	
Recombinant DNA		
pFA6a-HIS3-MX6	Longtine et al., 1998	
pSB031 (Hyg+-P_{MET25}-Kozak-start codon-AID*-6xFlag)	This study	
Sequence-Based Reagents		
A full list of DNA oligos is presented in Figure S5.		
Software and Algorithms		
pyCRAC	Webb et al., 2014	https://bitbucket.org/sgrann/pycrac
HISAT2 v2.02	Kim et al., 2015	https://ccb.jhu.edu/software/hisat2/index.shtml
SAMtools v1.3.1		http://www.htslib.org/
Bedtools v2.25		https://github.com/arq5x/bedtools2
featureCounts v1.4.2		http://bioinf.wehi.edu.au/featureCounts/
MEME		http://meme-suite.org/
Prism 7	Graphpad	www.graphpad.com
Integrative Genomics Viewer	Broad Institute	http://software.broadinstitute.org/software/igv/

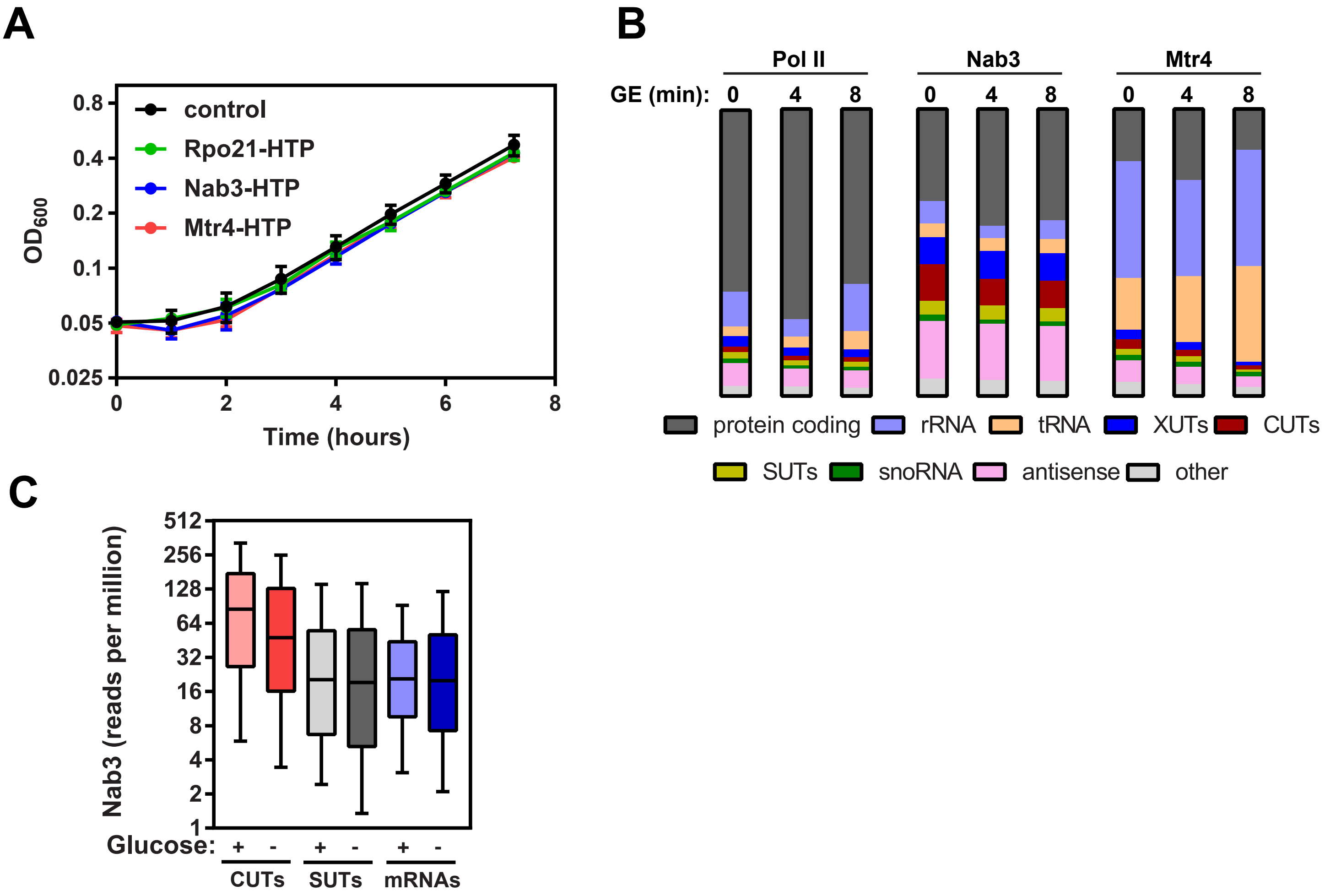
A
0.8
0.4
0.2
0.1
0.05
0.025
OD600
control
Rpo21-HTP
Nab3-HTP
Mtr4-HTP
0
2
4
6
8
Time (hours)
B
Pol II
Nab3
Mtr4
GE (min):
0
4
8
0
4
8
0
4
8
protein coding
rRNA
tRNA
XUTs
CUTs
SUTs
snoRNA
antisense
other
C
512
256
128
64
32
16
8
4
2
1
Nab3 (reads per million)
Glucose:
+
-
+
-
+
-
CUTs
SUTs
mRNAs

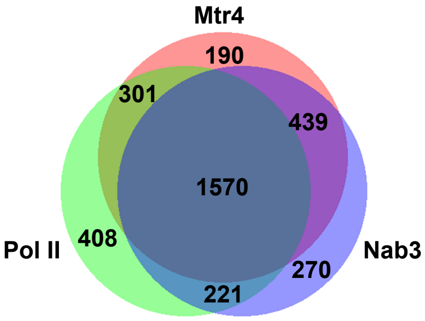
Mtr4
190
301
439
1570
Pol II
408
Nab3
270
221

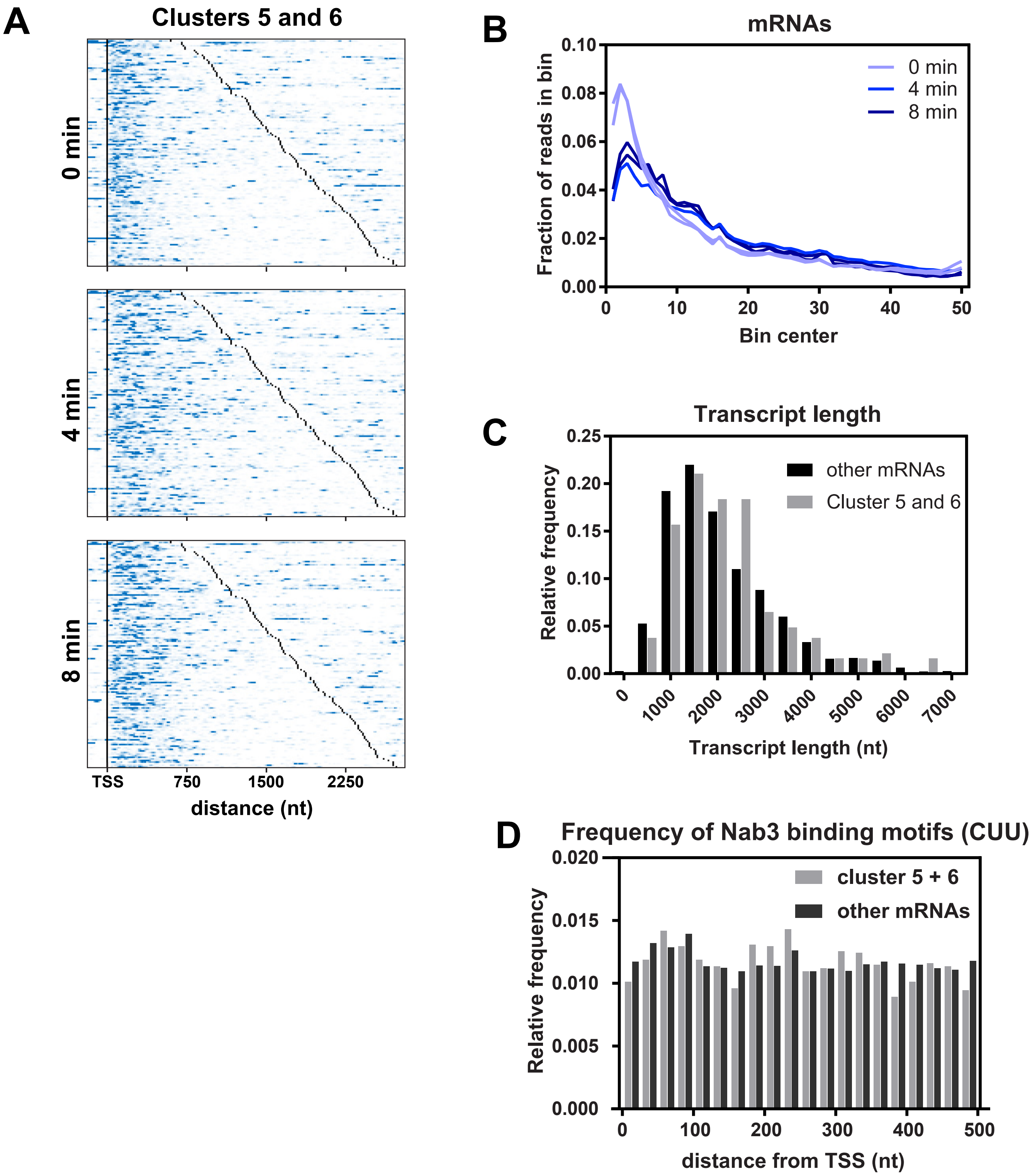
A
Clusters 5 and 6
0 min
4 min
8 min
TSS
750
1500
2250
distance (nt)
B
mRNAs
Fraction of reads in bin
0.10
0.08
0.06
0.04
0.02
0.00
0 min
4 min
8 min
0
10
20
30
40
50
Bin center
C
Transcript length
Relative frequency
0.25
0.20
0.15
0.10
0.05
0.00
other mRNAs
Cluster 5 and 6
0
1000
2000
3000
4000
5000
6000
7000
Transcript length (nt)
D
Frequency of Nab3 binding motifs (CUU)
Relative frequency
0.020
0.015
0.010
0.005
0.000
cluster 5 + 6
other mRNAs
0
100
200
300
400
500
distance from TSS (nt)

Bresson et al. Figure S4

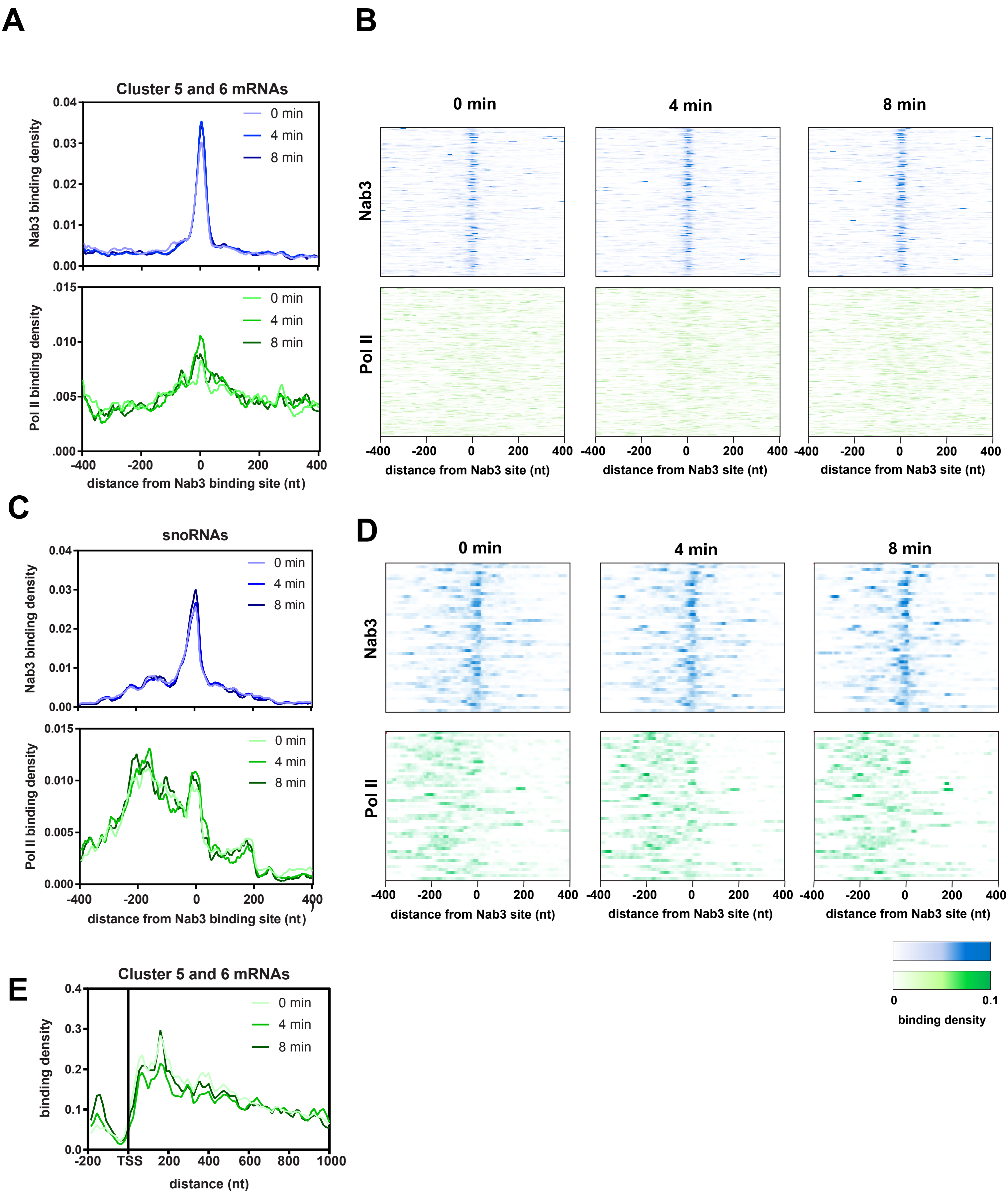

Bresson et al. Figure S5

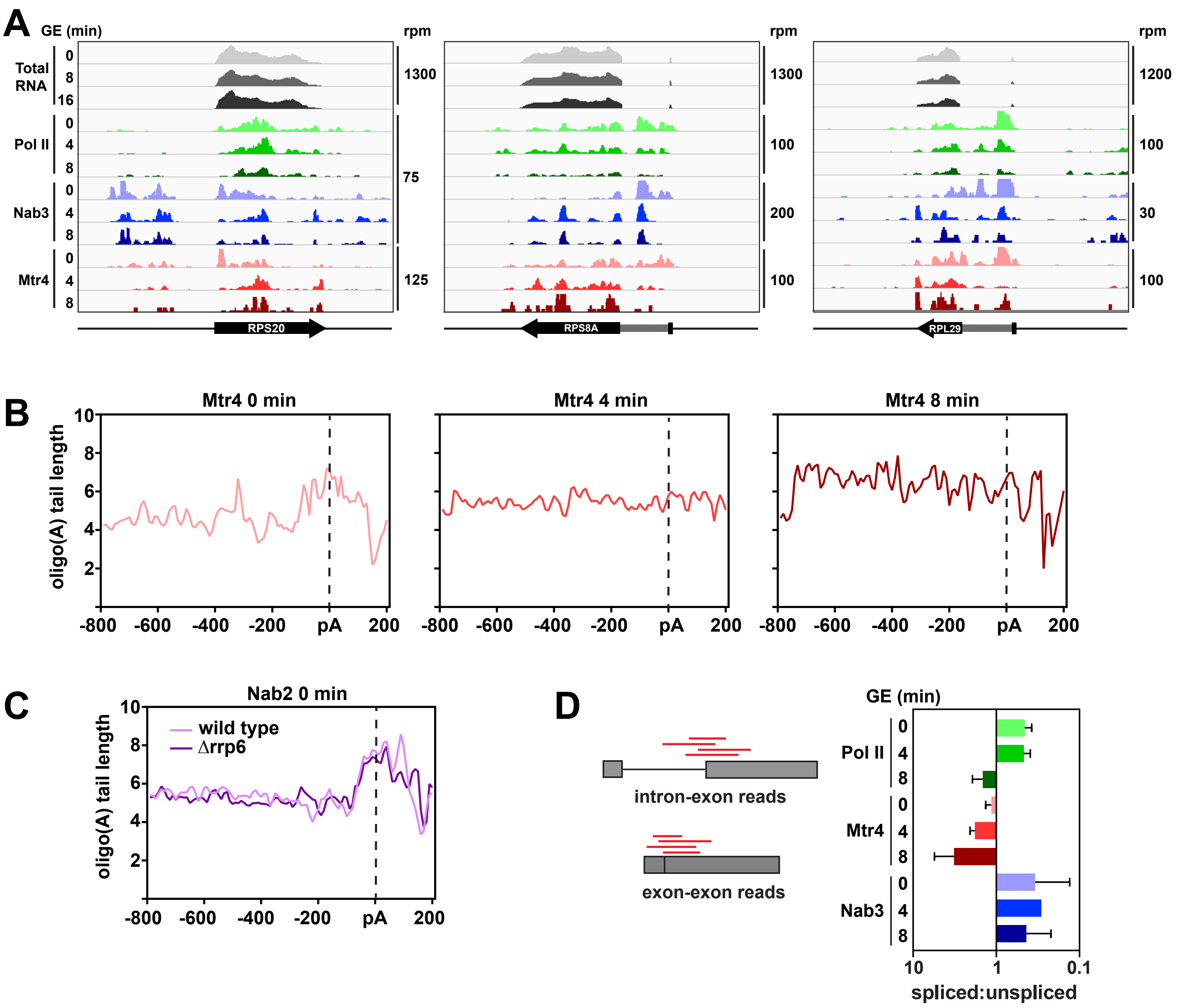

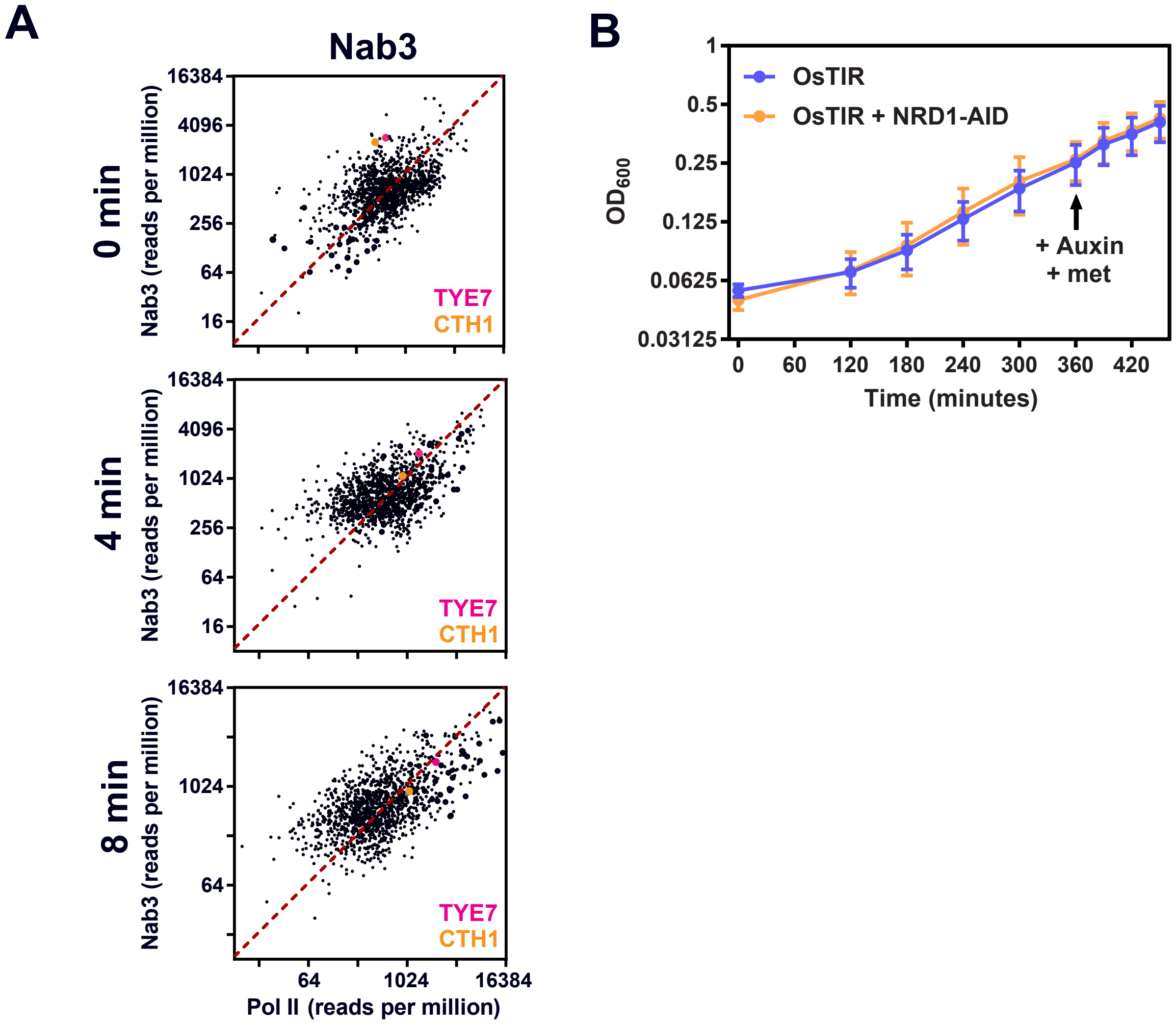
A
Nab3
0 min
4 min
8 min
Nab3 (reads per million)
16384
4096
1024
256
64
16
TYE7
CTH1
Pol II (reads per million)
B
OsTIR
OsTIR + NRD1-AID
OD600
1
0.5
0.25
0.125
0.0625
0.03125
+ Auxin
+ met
0
60
120
180
240
300
360
420
Time (minutes)
